# Blood Pressure Control and Maintenance in U.S. Veterans: Roles of Sex, Race, Ethnicity, and Deprivation

**DOI:** 10.1016/j.jacadv.2025.102267

**Published:** 2025-10-24

**Authors:** Allison E. Gaffey, Tiffany E. Chang, Sanket S. Dhruva, Matthew M. Burg, Sally G. Haskell, Lori A. Bastian, Erica S. Spatz, Allison Levine, Melissa Skanderson, Cynthia A. Brandt

**Affiliations:** a VA Connecticut Healthcare System, West Haven, Connecticut, USA; b Department of Internal Medicine (Cardiovascular Medicine), Yale School of Public Health, New Haven, Connecticut, USA; c Department of Psychiatry, Yale School of Medicine, New Haven, Connecticut, USA; d Department of Chronic Disease Epidemiology, Yale School of Public Health, New Haven, Connecticut, USA; e San Francisco Veterans Affairs Health Care System, San Francisco, California, USA; f Section of Cardiology, Department of Medicine, UCSF School of Medicine, San Francisco, California, USA; g Department of Anesthesiology, Yale School of Medicine, New Haven, Connecticut, USA; h Department of Internal Medicine (General Medicine), Yale School of Medicine, New Haven, Connecticut, USA; i Department of Biostatistics, Yale School of Medicine, New Haven, Connecticut, USA

**Keywords:** blood pressure, hypertension, race, sex differences, social determinants of health, veterans

## Abstract

**Background:**

U.S. Veterans have a greater prevalence and earlier onset of cardiovascular disease than non-Veterans. Thus, blood pressure (BP) control is particularly beneficial for younger Veterans, discharged after October 1, 2001 (ie, post-9/11).

**Objectives:**

The objective of the study was to assess BP control and maintenance by sociodemographic characteristics among post-9/11-era Veterans with new-onset hypertension.

**Methods:**

This retrospective cohort study included data from all post-9/11 Veterans who received care in U.S. Veterans Affairs medical centers, October 1, 2001-September 30, 2023 (n = 1,280,441). Hypertension was defined as the first diagnosis, antihypertensive fill, or ≥2 outpatient BP ≥140/90 mm Hg. Exposures were sex, race, and ethnicity. The Social Deprivation Index was calculated from zip codes. Logistic regression tested associations between sociodemographic variables and BP control (<140/90 mm Hg), 1, 2, and 5 years after hypertension onset, while covarying demographics, behavioral, and clinical factors.

**Results:**

Overall, 31% of patients met the hypertension criteria and had adequate follow-up (n = 398,732; median age: 37 years, 10% women, 63% non-Hispanic [NH] White). One year after onset, 43% of men and 60% of women achieved BP control, improving to 59% and 67% by 5 years. After adjustment, women had greater odds of control at 1 year (OR: 1.85; 95% CI: 1.81-1.90), which remained at 5 years (OR: 1.39; 95% CI: 1.34-1.44). Relative to NH White patients, Hispanic patients had 23% to 17% greater control odds, and NH Black patients had 10% to 23% lower odds through 5 years. Inclusion of the social Deprivation Index did not change these results.

**Conclusions:**

The first year of hypertension management portends future differences in BP control. Earlier strategies are needed to improve BP control in a population at high-risk for cardiovascular disease.

Guideline-based blood pressure (BP) management can mitigate the short- and long-term impact of hypertension on cardiovascular health and distal effects on the risk for cardiovascular disease (CVD).^1^ Men and women Veterans show earlier elevations in BP and a greater risk of hypertension and CVD than non-Veterans.^2^, ^3^, ^4^, ^5^, ^6^ Thus, early BP control could be particularly beneficial for younger U.S. military Veterans—that is, those who served in Iraq and Afghanistan conflicts and were discharged post-9/11.^7^ Half of younger Veterans seek care through the Department of Veterans Affairs (VA).^8^ As younger adults show lower hypertension awareness, treatment, and control than older adults,^9^ information about BP control in this at-risk group of Veterans can improve hypertension management and reduce CVD risk.

In the general population, non-Hispanic (NH) Black men and women show the greatest prevalence of hypertension,^1^^,^^10^ and hypertension management varies by sex, race, and ethnicity.^11^, ^12^, ^13^ Importantly, post-9/11 Veterans are younger and more demographically diverse than those who served earlier.^14^^,^^15^ By 2042, women will represent 20% of Veterans,^8^ and more than one-third of women identify as Black.^16^ Women Veterans are more likely to use VA benefits,^8^ and also receive less optimal cardiovascular care than men.^17^ Demographic differences in hypertension onset and BP control may also relate to social determinants of health (SDoH),^18^ which adversely affect the cardiovascular health of disadvantaged groups.^19^^,^^20^ Those with a lower income, who live in under-resourced neighborhoods, and who have less health care access are all less likely to achieve controlled BP.^21^ Understanding if there are demographic differences in BP control and maintenance among post-9/11 Veterans is necessary to support the cardiovascular health care needs of those served in VA and non-VA settings.

This retrospective cohort study used VA electronic health records (EHRs) from all post-9/11 Veterans to pursue three objectives. First, to establish the proportion of patients who met BP control at 1, 2, and 5 years after hypertension onset by sex, race, and ethnicity. Second, to determine if there were differences in BP control based on sex, race, and ethnicity. We hypothesized that BP control would increase over patients’ follow-up time, particularly for women and non-White patients compared to other subgroups. A third objective was to test if including a composite measure of SDoH would affect subgroup differences in BP control.

## Methods

### Data sources

Data used for these analyses are covered by a proprietary data use agreement with the VA and are not available for distribution. Eligible men and women were identified from rosters maintained by the Department of Defense’s Manpower Data Center-Contingency Tracking System. We extracted patients’ EHRs from the national VA Corporate Data Warehouse. EHR included vital sign and coded diagnostic and procedural data from VA health care visits, combined with additional administrative, pharmacy, and diagnostic data (using International Classification of Diseases, Ninth Revision, Clinical Modification and Tenth Revision] codes and dates). This study was approved by the VA Connecticut Institutional Review Board with a waiver of informed consent. This paper follows the Strengthening the Reporting of Observational Studies in Epidemiology guidelines (see Supplemental Appendix).

### Study population

The Women Veterans Cohort Study is a retrospective investigation of all post-9/11 men and women Veterans (ie, who served during U.S. conflicts in Iraq and Afghanistan [Operations Enduring Freedom, Iraqi Freedom, and New Dawn]), who were discharged from service as of October 1, 2001, and whose first VA outpatient medical visit occurred before September 30, 2023.^14^^,^^15^ The cohort was restricted to those meeting the criteria for hypertension based on any one of the following: 1) an International Classification of Diseases-9th or 10th Revision code recorded during at least 2 outpatient encounters; 2) ≥1 antihypertensive medication prescription filled at a VA Pharmacy; and/or 3) ≥2 systolic outpatient BP ≥140 mm Hg, or ≥2 diastolic outpatient BP of ≥90 mm Hg on 2 separate days.^22^ Medication classes included ACE inhibitors, ARBs, beta-blockers, calcium channel blockers, diuretics, central alpha-2 agonists, alpha-1 blockers, vasodilators, aldosterone antagonists, and direct renin inhibitors. Eligible patients had ≥2 qualifying BP taken after their first date of VA care and before the end of their 5-year follow-up. To explore differences by SDoH, patients were excluded if they had a missing or invalid 5-digit residential zip code, or if their zip code corresponded to Puerto Rico, as SDoH data were unavailable (n = 7,311).

### Primary and secondary predictors: Sex, race, and ethnicity

Patients’ sex (men and women), race, and ethnicity (NH White, NH Black, Hispanic, NH Asian, and NH other race) were extracted from the Department of Defense roster files. Sex was the primary predictor. Race and ethnicity were combined as the secondary predictor due to distinctions in cardiovascular risk between these subgroups.

### Exploratory predictor: Social deprivation index

An index of SDoH was used to explore if inclusion influenced associations between demographics and likelihood of BP control. The Social Deprivation Index (SDI) is a composite of a geographic area’s estimated deprivation level that is derived from 7 measures in the American Community Survey: percentage of adults living in poverty; <12 years education; residing in a single-parent household, a rented housing unit, or an overcrowded housing unit; households without a car; and unemployed individuals aged <65 years.^23^ Scores were linked with patients’ residential zip codes. Scores range from 1 to 100, with higher scores indicating greater disadvantage. SDI was classified into quartiles (quartile 1 = least disadvantaged to quartile 4 = most disadvantaged).

### Outcome: BP control

BP control was assessed using the last 2 outpatient clinic systolic and diastolic BP measurements closest to 1, 2, and 5 years after a patient first met the criteria for hypertension. BPs were limited to outpatient settings to avoid potential transient changes during hospitalizations.^24^ See the Supplemental Material for additional BP selection details. As most data were collected before the 2017 update to hypertension guidelines,^25^ BP control was defined as <140/90 mm Hg for primary analyses.

### Covariates

Based on earlier analyses of factors associated with incident hypertension and BP control in Veterans, women, and the general population,^6^^,^^11^^,^^13^^,^^26^, ^27^, ^28^, ^29^, ^30^ a priori statistical covariates included demographics: age (<40 vs ≥40 years) and marital status (married vs not married); behavioral factors: smoking status (current, past, never), body mass index (categories of underweight/normal, overweight, and obese [reference]), and history of alcohol or substance use disorder (ever diagnosed vs never [reference]); history of military sexual trauma (MST) (any report vs not reported [reference]^27^^,^^30^) and other clinical risk factors (ever diagnosed vs never [reference]): post-traumatic stress disorder (PTSD), major depressive disorder (MDD), generalized anxiety disorder (GAD), diabetes, dyslipidemia, and obstructive sleep apnea (OSA); and the number of comorbidities using a modified Charlson Comorbidity Index (ie, excluding leukemia and lymphoma in the weights).^31^ Lastly, a health care utilization variable was created based on the number of primary care visits in the first 2 years.^32^ Covariates were measured at baseline (demographics, smoking status, body mass index, and history of MST) or by the first diagnosis (substance use and clinical risk factors). As rurality is included in SDI calculation, this variable was not examined as a covariate. For descriptive purposes, patients were also characterized by rurality of residence, availability of additional non-VA health insurance at baseline, baseline BP category, and if antihypertensive medication was prescribed to a patient, by the first antihypertensive that was prescribed.

### Statistical analysis

Analyses were conducted using SAS software version 9.4 (SAS Institute, Inc) and *P* < 0.05. First, descriptive and bivariate statistics were used to summarize the data and to determine differences in BP control at 1, 2, and 5 years by demographic factors. Next, random intercept, multilevel logistic regression models were conducted using the GLIMMIX procedure to assess the relations between sex, race and ethnicity, and BP control. BP control odds were modeled separately for 1, 2, and 5 years. Since the primary predictors were sex, race, and ethnicity, initial, unadjusted models only included sex as the independent predictor (model 1). Next, logistic regression models included stepwise adjustment for: other demographics (model 2), behavioral factors (model 3), and clinical factors (model 4). We then tested sex∗race/ethnicity interactions on BP control, followed by race/ethnicity and sex-stratified analyses. SDI was added as a predictor in exploratory analyses. Finally, as hypertension categories changed in 2017,^25^ sensitivity analyses were limited to 2018 to 2023 data, using 130/80 mm Hg for hypertension and BP control thresholds.

## Results

**Figure 1:**
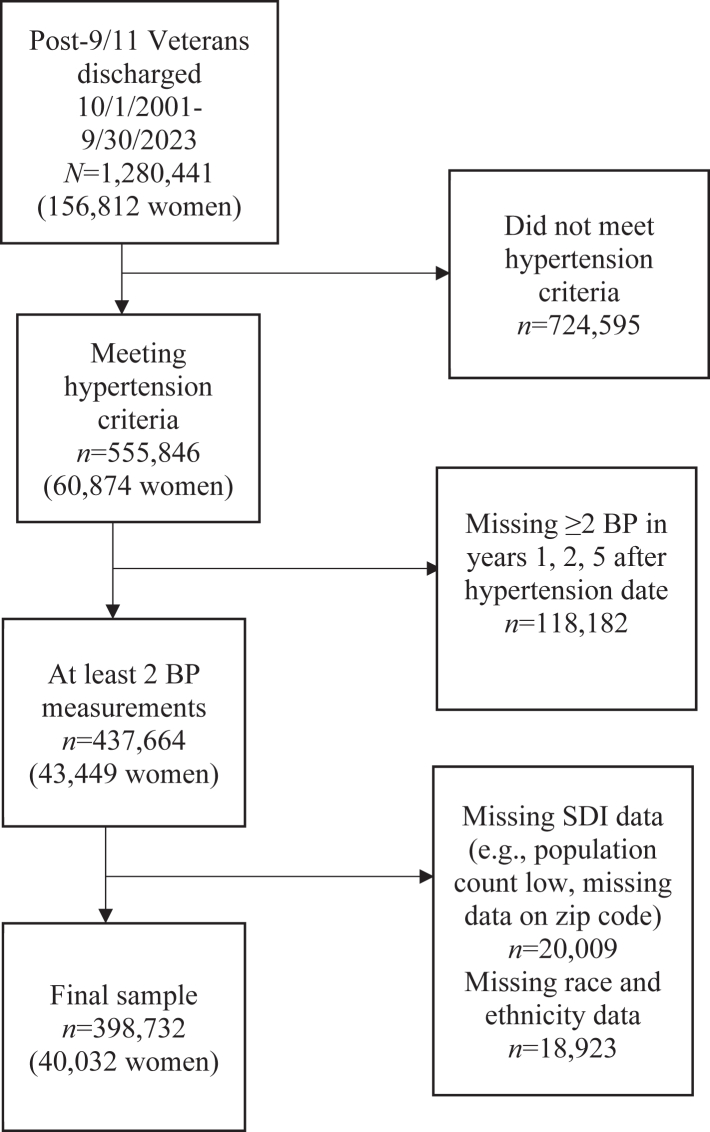
Study Flowchart of the Retrospective Cohort of Post-9/11 U.S. Veterans The cohort was based on all Veterans who were discharged and initiated care with the Veterans Health Administration, October 1, 2001-September 30, 2023 (ie, post-9/11). The analytic sample was limited to those who met criteria for hypertension based on first diagnosis, antihypertensive fill, or ≥2 outpatient blood pressure (BP) readings ≥140/90 mm Hg and who had sufficient sociodemographic data, including to calculate the Social Deprivation Index (SDI).

Data were available for 1,280,441 patients (12% women) (Figure 1). Overall, 43% of patients met at least 1 hypertension criteria (555,846, 11% women) and 31% also had adequate demographic and follow-up data (398,732, 10% women, 63% NH White) (Supplemental Table 1). Men and women were 37 and 38 years old at baseline (Table 1). Relative to men, a significantly greater percentage of women were non-White, unmarried, and lived in an urban locale. Proportions of men and women were similar across SDI quartiles. Among behavioral factors, more men than women had ever smoked, were obese, or had a substance use disorder history. In relation to men, a greater percentage of women had a documented history of MST, but significantly more men than women had PTSD, diabetes, dyslipidemia, or OSA Women had greater primary care utilization, but follow-up time was about 5 years for each group. Characteristics were also described within sex, by race and ethnicity (Supplemental Tables 2 and 3).

**Table 1:** Demographic, Behavioral, and Clinical Factors Presented by Sex (N = 398,732)

	Men (n = 358,700)	Women (n = 40,032)	*P* Value
Age, y	37.0 (30,46)	38.1 (31,46)	<0.001
<40	210,626 (58.7)	22,506 (56.2)	
≥40	148,074 (41.3)	17,526 (43.8)	
Race and ethnicity			<0.001
NH White	234,381 (65.3)	**17,964 (44.9)**	
NH Black	67,448 (18.8)	**15,800 (39.5)**	
Hispanic	37,203 (10.4)	**3,793 (9.5)**	
NH Asian	8,646 (2.4)	**896 (2.2)**	
NH other	11,022 (3.1)	**1,579 (3.9)**	
Married	200,757 (56.4)	15,066 (38.0)	<0.001
Rural	102,767 (28.7)	8,403 (21.0)	0.197
Additional health insurance	150,379 (42.0)	16,651 (41.7)	<0.001
Social deprivation			<0.001
Quartile 1 (least disadvantaged)	92,899 (25.9)	8,973 (22.4)	
Quartile 2	89,038 (24.8)	9,717 (24.3)	
Quartile 3	87,915 (24.5)	10,080 (25.2)	
Quartile 4 (most disadvantaged)	88,848 (24.8)	11,262 (28.1)	
Behavioral/lifestyle factors			
Current smoker	153,160 (43.0)	10,691 (26.8)	<0.001
Obese	193,979 (55.4)	19,235 (49.0)	<0.001
Substance use disorder	105,260 (29.3)	7,517 (18.8)	<0.001
Clinical factors			
Military sexual trauma	7,967 (2.3)	13,347 (33.8)	<0.001
PTSD	205,948 (57.4)	22,395 (55.9)	<0.001
MDD	143,977 (40.1)	22,834 (57.0)	<0.001
GAD	37,962 (10.6)	7,181 (17.9)	<0.001
Diabetes	53,207 (14.8)	4,865 (12.2)	<0.001
Dyslipidemia	178,423 (49.7)	14,217 (35.5)	<0.001
OSA	131,514 (36.7)	9,255 (23.1)	<0.001
Number of comorbidities			<0.001
0	139,871 (39.0)	12,596 (31.5)	
1	127,753 (35.6)	16,252 (40.6)	
2	48,171 (13.4)	6,648 (16.6)	
≥3	42,905 (12.0)	4,536 (11.3)	
Health care utilization			<0.001
# Primary care visits	5 (3,8)	7 (4,11)	<0.001
Follow-up period	5.5 (2,10)	5 (2,9)	<0.001
Baseline BP category			<0.001
≥160/100 mm Hg	24,887 (6.9)	1,583 (4.0)	
140-159/90-99 mm Hg	101,240 (28.2)	6,509 (16.3)	
130-139/80-89 mm Hg	135,007 (37.6)	13,218 (33.0)	
120-129/<80 mm Hg	47,230 (13.2)	5,557 (13.9)	
<120/80 mm Hg	50,336 (14.0)	13,165 (32.9)	
First AHM category prescribed			<0.001
ACE inhibitor	57,428 (16.0)	3,344 (8.4)	
ARB	12,584 (3.5)	942 (2.4)	
Beta-blocker	64,513 (18.0)	11,025 (27.5)	
Calcium channel blocker	34,208 (9.5)	4,176 (10.4)	
Diuretic	39,482 (11.0)	8,016 (20.0)	
Diuretic combo with other class	19,680 (5.5)	1969 (4.9)	
Other class combo (no diuretic)	759 (0.2)	64 (0.2)	
Other medications	13,565 (3.8)	1,417 (3.5)	

Values are N (%) or median (Q1, Q3). *P* value tests differences across all sex, race, and ethnicity groups using chi-square test for categorical variables, Kruskal-Wallis test for continuous non-normally distributed variables. If an individual was in 2 distinct categories based on systolic and diastolic BP, they were placed in the higher BP category. Other medications were vasodilators, direct renin inhibitors, and central α_2_-2 antagonists.

ACE inhibitor = angiotensin-converting enzyme inhibitor; AHM = antihypertensive medication; ARB = angiotensin receptor blocker; BP = blood pressure; GAD = generalized anxiety disorder; MDD = major depressive disorder; NH = non-Hispanic; OSA = obstructive sleep apnea; PTSD = post-traumatic stress disorder.

### Predictors of BP control and maintenance

In analyses of BP control and maintenance by sex, more women than men achieved BP control at each time point (year 1: 60% vs 43%, year 2: 70% vs 59%, year 5: 67% vs 59%) (Supplemental Table 4). In unadjusted results for year 1, women had 1.99 times greater odds of control (95% CI: 1.94-2.03), which decreased to 1.85 times greater odds (95% CI: 1.81-1.90) (Table 2) after adjusting for demographic, behavioral, and clinical factors. Younger age, Hispanic and Asian demographics, and a diagnosis of PTSD were also associated with increased odds of BP control after 1 year, but the NH Black race and ethnicity group, unmarried status, current smoking, overweight or obese weight categories, and a substance use history were associated with lower odds. At years 2 and 5, women had 1.60 and 1.41 greater unadjusted odds of BP control (95% CIs: 1.56-1.64 and 1.34-1.44), and 1.55 and 1.39 greater odds after adjustment (95% CIs: 1.50-1.60 and 1.34-1.44).

**Table 2:** Multivariable Models of BP Control at 1-5 Years, by Sex

	Year 1		Year 2		Year 5	
	OR (95% CI)	*P* Value	OR (95% CI)	*P* Value	OR (95% CI)	*P* Value
Women	1.85 (1.81-1.90)	<0.001	1.55 (1.50-1.60)	<0.001	1.39 (1.34-1.44)	<0.001
Demographics						
<40 y (vs ≥40 y)	1.09 (1.07-1.10)	<0.001	0.99 (0.97-1.00)	0.102	0.97 (0.95-0.99)	0.002
NH Black (vs NH White)	0.90 (0.88-0.91)	<0.001	0.82 (0.80-0.84)	<0.001	0.77 (0.75-0.79)	<0.001
Hispanic (vs NH White)	1.07 (1.04-1.09)	<0.001	1.11 (1.08-1.14)	<0.001	1.07 (1.04-1.11)	<0.001
NH Asian (vs NH White)	1.09 (1.04-1.14)	0.001	0.99 (0.94-1.05)	0.833	1.02 (0.95-1.09)	0.607
NH other (vs NH White)	0.94 (0.90-0.98)	<0.001	0.94 (0.89-0.98)	0.006	0.98 (0.93-1.04)	0.445
Not married (vs married)	0.89 (0.87-0.90)	<0.001	0.91 (0.90-0.93)	<0.001	0.90 (0.87-0.91)	<0.001
Behavioral/lifestyle factors						
Past smoker (vs never)	0.88 (0.87-0.90)	0.030	0.90 (0.88-0.91)	<0.001	0.91 (0.89-0.93)	<0.001
Current smoker (vs never)	0.98 (0.96-1.00)	<0.001	0.99 (0.97-1.01)	<0.001	1.01 (0.98-1.04)	0.583
Overweight (vs normal/under)	0.78 (0.76-0.80)	<0.001	0.77 (0.75-0.79)	<0.001	0.80 (0.77-0.83)	<0.001
Obese (vs normal/under)	0.63 (0.62-0.65)	<0.001	0.61 (0.59-0.63)	<0.001	0.64 (0.62-0.66)	<0.001
Substance use disorder (vs never)	0.94 (0.92-0.95)	<0.001	0.86 (0.84-0.87)	<0.001	0.85 (0.84-0.87)	<0.001
Clinical risk factors						
# Of comorbidities	1.03 (1.03-1.04)	<0.001	0.99 (0.98-0.99)	<0.001	0.99 (0.98-0.99)	0.039
PTSD ever (vs never)	1.19 (1.18-1.21)	<0.001	1.08 (1.06-1.10)	<0.001	1.06 (1.04-1.08)	<0.001
OSA ever (vs never)	1.03 (1.02-1.03)	<0.001	1.00 (1.00-1.00)	0.170	1.01 (1.00-1.01)	0.360
Diabetes (vs none)	1.09 (1.07-1.10)	<0.001	0.99 (0.97-1.01)	0.006	0.95 (0.93-0.97)	<0.001
Dyslipidemia (vs none)	1.05 (1.03-1.08)	<0.001	0.97 (0.94-0.99)	0.566	0.99 (0.96-1.02)	0.661
MST ever (vs no history)	1.08 (1.05-1.12)	<0.001	1.05 (1.01-1.09)	0.013	1.04 (0.99-1.09)	0.126
Primary care utilization						
# Of visits in the first 2 years	1.06 (1.04-1.08)	<0.001	1.00 (0.98-1.01)	<0.001	1.00 (0.98-1.02)	0.844

MST = military sexual trauma; other abbreviations as in Table 1.

Table 3 presents multivariable models stratified by sex, race, and ethnicity. Sex∗race interactions were significant at each follow-up year (*P* < 0.001). In unadjusted analyses, women in all race and ethnic groups showed a greater odds of BP control over time than men (ORs: year 1: 1.50-2.29, year 2: 1.34-1.79, year 5: 1.39-1.62), although there was no significant difference for Asian patients at year 5 (*P* = 0.725). After adjustment, women from all race and ethnic groups showed greater BP control than men from their groups (ORs: year 1: 1.26-2.07, year 2: 1.22-1.63, year 5: 1.13-1.43) (Supplemental Tables 5 to 7), except for Asian men and women at years 2 and 5 (*P* = 0.071 and 0.343) and other men and women at year 5 (*P* = 0.212). Next, race and ethnic groups were compared within each sex (Supplemental Table 8). Compared to NH White men, NH Black patients had consistently worse BP control over time (year 1: OR: 0.96; 95% CI: 0.94-0.98, year 2: OR: 0.86 95% CI: 0.84-0.88, year 5: OR: 0.79; 95% CI: 0.77-0.81), while Hispanic patients had better control than White men during the same period (year 1: OR: 1.09; 95% CI: 1.06-1.11, year 2: OR: 1.13; 95% CI: 1.10-1.17, year 5: OR: 1.09; 95% CI: 1.05-1.13). Compared to NH White women, NH Black, Asian, and Other race women showed lower BP control odds 1 to 5 years later, whereas Hispanic women had better control at year 1 only (OR: 1.14; 95% CI: 1.05-1.23; Central Illustration). Adding SDI did not change odds of BP control by sex, race, or ethnicity, and the effect of SDI was small (Supplemental Table 9).

**Table 3:** Multivariable Models of BP Control at 1-5 Years, by Sex, Race, and Ethnicity

Within Sex by Race and Ethnicity	OR (95% CI)	*P* Value
Year 1		
NH White (Women vs Men)	1.96 (1.89-2.04)	<0.001
NH Black	1.74 (1.67-1.81)	<0.001
Hispanic	2.07 (1.90-2.26)	<0.001
NH Asian	1.26 (1.07-1.49)	<0.001
NH Other	1.78 (1.56-2.04)	<0.001
Year 2		
NH White (Women vs Men)	1.63 (1.56-1.70)	<0.001
NH Black	1.48 (1.41-1.56)	<0.001
Hispanic	1.61 (1.45-1.78)	<0.001
NH Asian	1.22 (0.99-1.50)	0.067
NH Other	1.58 (1.35-1.86)	<0.001
Year 5		
NH White (Women vs Men)	1.47 (1.40-1.56)	<0.001
NH Black	1.38 (1.30-1.46)	<0.001
Hispanic	1.43 (1.26-1.62)	<0.001
NH Asian	0.88 (0.68-1.14)	0.340
NH Other	1.13 (0.93-1.39)	0.215

Within race and Ethnicity by Sex	Men	Women
	OR (95% CI)	OR (95% CI)
Year 1		
NH Black (vs NH White)	0.96 (0.94-0.98)	0.84 (0.80-0.88)
Hispanic (vs NH White)	1.09 (1.06-1.11)	1.14 (1.05-1.23)
NH Asian (vs NH White)	1.14 (1.09-1.19)	0.78 (0.67-0.90)
NH Other (vs NH White)	0.97 (0.93-1.01)	0.86 (0.76-0.95)
Year 2		
NH Black (vs NH White)	0.86 (0.84-0.88)	0.77 (0.72-0.81)
Hispanic (vs NH White)	1.13 (1.10-1.17)	1.08 (0.98-1.19)
NH Asian (vs NH White)	1.03 (0.97-1.09)	0.76 (0.63-0.91)
NH other (vs NH White)	0.95 (0.91-1.00)	0.87 (0.76-1.00)
Year 5		
NH Black (vs NH White)	0.79 (0.77-0.81)	0.70 (0.66-0.75)
Hispanic (vs NH White)	1.09 (1.05-1.13)	1.04 (0.93-1.17)
NH Asian (vs NH White)	1.07 (1.00-1.14)	0.67 (0.54-0.84)
NH other (vs NH White)	1.01 (0.96-1.08)	0.79 (0.67-0.94)

Abbreviations as in Table 1.

**Central Illustration:**
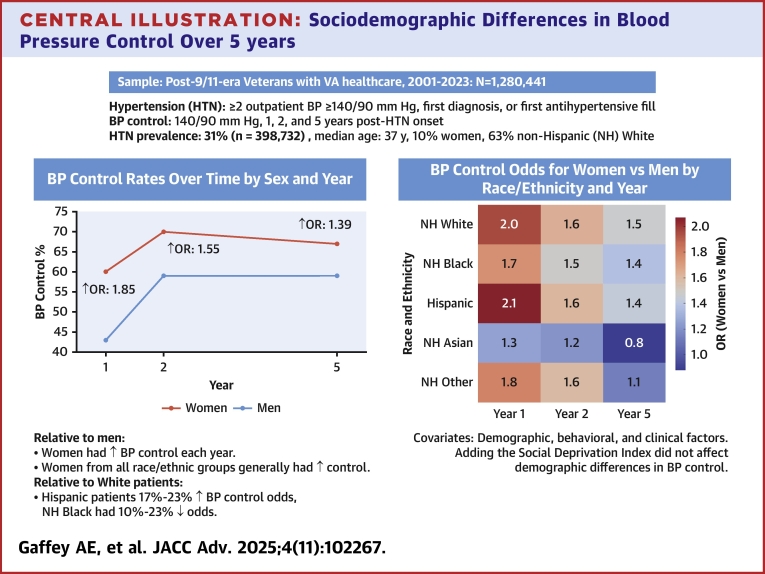
Sociodemographic Differences in Blood Pressure Control Over 5 years BP = blood pressure; VA = Department of Veterans Affairs.

### BP control with 130/80 mm Hg

Sensitivity analyses were conducted using 2018 to 2023 data and 130/80 mm Hg as the hypertension threshold. With these conditions, 152,859 patients met the criteria for hypertension (median age of 39 years, 13% women, 62% NH White) (Supplemental Tables 10 and 11). In year 1, 13% of men and 26% of women achieved BP control, which increased over time for each group (year 2: 27% and 38%, year 5: 25% and 35%) (Supplemental Table 12). In unadjusted analyses, women had greater odds of BP control at years 1 to 5 (ORs: 1.56-2.77). After adjustment, women had 2.42 better odds than men at year 1 (95% CI: 2.32-2.53) (Supplemental Table 13), 1.61 better odds of BP control at year 2 (95% CI: 1.53-1.70), and 1.41 better odds at year 5 (95% CI: 1.30-1.54). Younger age, smoking, and obesity were associated with worse BP control at each time. Sex∗race interactions were significant at each follow-up year (*P*s < 0.001). Women in each race and ethnicity group had better BP control than men from the same group at each time (ORs: year 1: 2.01-2.99, year 2: 1.12-2.11) (Supplemental Table 14). Hispanic men and women consistently had greater odds of BP control than same-sex Whites (ORs: 1.17-1.23). Relative to NH White patients, NH Black men and women showed no difference in BP control at year 1, but their odds of control were significantly lower at years 2 and 5 (ORs: 0.94 and 0.84). Again, adding the SDI did not significantly change these associations (Supplemental Table 15).

## Discussion

In this retrospective cohort study of all post-9/11 Veterans, 43% met the initial criteria for hypertension, and 31% also had adequate follow-up data. Within 1 year, 43% of men and 60% of women had controlled BP. Although only 60% of men and 67% of women achieved BP control at 5 years, women had consistently greater BP control over time. Relative to NH White patients, Hispanic men and women each showed better BP control and NH Black men and women had worse control. All relations were consistent after adding SDI, suggesting that demographic differences persist despite variations in some SDoH and the benefits of accessing VA care. When using a lower hypertension threshold (ie, 130/80 mm Hg), a minority of patients had controlled BP at 1 year or thereafter. Importantly, difficulty achieving hypertension goals is also observed in the general U.S. population, and BP control has recently declined across all demographic groups.^8^^,^^9^ Therefore, although the post-9/11 Veteran population has unique health risk factors and benefits from access to the VA system of care, the group appears to experience similar challenges with short- and long-term BP management.

In the United States, the prevalence of hypertension among young and middle-aged adults is estimated as 31% to 59% for men and 13% to 50% for women,^29^^,^^33^^,^^34^ statistics which are like the present results. Overall, women consistently showed better BP control than men, indicating that VA care appeared to be more effective for women Veterans. Still, women may experience later barriers to managing their cardiovascular health. For example, women cared for in the VA system are less likely to receive a CVD diagnosis and guideline-directed management for established CVD than men,^17^ and inequality in secondary CVD prevention has also been reported in non-VA settings.^12^ Although no comparable study has examined sex differences in BP control among Veterans receiving care through the VA, studies in the general population have reported mixed results, with observed differences varying by age, race, and ethnicity.^11^^,^^29^^,^^35^^,^^36^ Sex differences in Veterans’ hypertension management may begin with patients’ health-related knowledge. Younger women are 35% more likely to know that they have hypertension than same-aged men,^37^ awareness that may relate to greater healthcare utilization in women Veterans and non-Veterans.^32^^,^^38^ Sex differences in BP control could also reflect other differences in health care knowledge and utilization,^7^^,^^15^ including use of both VA and community (ie, non-VA) care. To improve short-term BP control, sex-specific approaches to hypertension management may be helpful, but approaches need to be developed and tested.^39^^,^^40^

When concurrently examining sex, race, and ethnicity, NH White men and women had greater odds of controlled BP than NH Black patients over time but worse control than Hispanic patients through 5 years. Our findings extend prior studies in Veterans^41^, ^42^, ^43^—examining sex by race/ethnicity interactions and comparing racial and ethnic subgroups within men and women. Given that the VA system is designed to offer standardized care to all Veterans, any disparities in BP management may alternately occur due to variance in treatment initiation or intensification, hypertension severity, clinician judgment and bias, and the quality of care or health care utilization at VA facilities frequented more by racial and ethnic minority groups.^44^^,^^45^ As an example, among 16,114 patients with hypertension from the general population, missed appointments reflected 13% to 14% of race differences in BP control.^46^ Some of the present results correspond with racial disparities observed in the National Health and Nutrition Examination Survey,^36^ in which NH Black participants had worse control than NH White participants.^36^

NH Black Veterans may have worse BP control for several reasons. First, even within the VA system, there may be disparities in access to high-quality, continuous, and culturally competent care. Black Veterans may be more likely to face socioeconomic barriers such as unstable housing, food insecurity, lower income, and neighborhood stressors, all of which can negatively affect BP control.^47^^,^^48^ A second potential reason is medical mistrust and communication barriers, which are well-documented and can impact Black patients’ adherence to medical recommendations and follow-up care.^46^^,^^49^ Implicit bias or poor communication between providers and patients may also lead to less effective treatment plans or lower patient engagement. A third reason relates to differences in treatment and medication and prescribing patterns. Black patients may be less likely to be prescribed guideline-concordant therapies, or could have delays in medication intensification.^46^ Relatedly, certain classes of antihypertensive medications (eg, angiotensin-converting enzyme [ACE] inhibitors) may be less effective in some Black patients compared to others, and this may not always be accounted for in prescribing decisions.^46^

In contrast, Hispanic patients in the present study had better BP control than other subgroups. Despite frequently showing a greater prevalence of traditional cardiovascular risk factors (eg, obesity, diabetes, and limited access to care) Hispanic individuals often have health outcomes that are paradoxically better than expected given their socioeconomic disadvantages, including among Veterans,^50^^,^^51^ a controversial phenomenon dubbed the "Hispanic Paradox.”^52^^,^^53^ Theories about the improved cardiovascular health outcomes for Hispanic Veterans include that Hispanic individuals have healthier diets and that recent and first generation immigrants have less substance use.^52^ It has also been suggested that strong family ties and community networks within Hispanic populations may offer additional support for health-promoting behaviors.^53^ Broadly, subgroup variations in BP control may also relate to differences in trust, awareness, adherence, and health beliefs,^13^ which have been highlighted as important in the general population and should be investigated in relation to Veterans’ hypertension management.^45^

SDoH can influence vulnerability to hypertension, treatment access, and long-term BP management,^17^ particularly among women.^46^ However, in this analysis, incorporating the SDI did not significantly alter associations between demographic factors and BP control. One explanation may lie in the context of the VA health care system—the largest integrated health system in the United States—which is designed to provide a uniform standard of care to Veterans and access to services like low-cost medications and transportation that supersedes social disadvantages, all of which can mitigate some of the health care inequities observed in the U.S. population.^54^ Although this health care system may buffer against certain SDoH-related barriers, it is likely insufficient to fully counteract demographic disparities in hypertension prevalence and control. Indeed, among Veterans receiving VA care, disparities related to sex, race, and ethnicity may subsume much of the variation that is otherwise captured by broader social factors.

The limited influence of the SDI on study outcomes may also reflect methodological and conceptual constraints.^55^ First, neighborhood-level assessments may not adequately capture individual-level drivers of BP control. Clinical and behavioral factors such as medication adherence, comorbidities, lifestyle, income, and trust in providers are more proximal and directly influential on BP management, particularly adherence to BP medications.^13^ Moreover, neighborhood-level indices like the SDI rely on aggregate data—typically at the zip code or Census tract level—which may not reflect the lived experiences or socioeconomic variation within those areas. Second, the SDI’s reliance on broad geographic units could mask subtle or localized disparities. In heterogeneous or mixed-income neighborhoods, socioeconomic averaging can obscure pockets of deprivation and relevant SDoH at the individual level.^56^ Finally, composite indices such as the SDI may not fully capture key contextual and psychosocial determinants of BP control. Although the SDI includes variables like income, education, and housing, these approaches often omit critical contextual factors—such as structural racism, community violence, cultural mistrust, psychosocial stress, and discrimination—that can profoundly impact health behaviors and treatment engagement.^57^^,^^58^ For example, in a multiethnic cohort study, neighborhood deprivation was associated with hypertension incidence but did not fully account for racial disparities in BP control.^59^ Therefore, although neighborhood-level SDoH measures offer valuable context, such measures may be insufficient to solely predict individual health outcomes like BP control.^60^ Future studies could benefit from a more nuanced, multilevel approach—integrating both neighborhood and individual-level data—to better understand and address disparities, particularly among post-9/11 Veterans. Investigating if specific components of deprivation (eg, education, employment, or housing) have stronger associations with BP outcomes, and whether these relations vary by demographic subgroup or severity of disadvantage, could provide more actionable insights.

### Study limitations

Limitations of this investigation should be enumerated. First, the sample consisted of only post-9/11 Veterans who sought VA care, so results are not generalizable to service members from earlier eras, those who do not receive VA care, or to the general population. For example, in relation to SDI, there could be misattribution if a patient’s true lived environment differs from their address on record (eg, homelessness, living between households). Second, as this was a retrospective cohort study involving EHR data, there is risk for misclassification bias, selection bias, and missing data. Third, it was not possible to control for all social, behavioral, and clinical factors. Among these limitations, medication adherence and length of use are particularly important. Although those data were unavailable, they will be valuable to include in subsequent investigations of demographic differences in BP control to understand how timing of BP agents may vary by group. Future investigations are also required to account for physical activity, diet, sleep duration, and psychosocial stress, as those factors show differential associations with hypertension by sex, race, ethnicity. Fourth, clinic BP measurement often does not reflect BP indexed in nonclinical settings. Ambulatory or home BP monitoring are recommended to confirm a diagnosis of hypertension and monitoring BP control but could not be captured in our data. Fifth, examining demographic differences in BP control by region, by type of antihypertensive used, among those taking monotherapy vs combination therapy, or clinician differences in BP management (eg, if initial antihypertensives are prescribed by a primary care physician vs a cardiologist or other specialty clinician), were all beyond the scope of the present analyses, and these questions are valuable for future investigations.^61^ Sixth, as the CHOICE and MISSION Acts encouraged Veterans to receive non-VA care,^62^^,^^63^ patients could have received hypertension care or antihypertensives outside of the VA system, which was beyond the scope of our analyses and therefore, could not be accounted for. Additional research is required to compare Veterans’ BP control between those treated exclusively by VA providers and those also receiving community care.

Although BP control is a key aspect of CVD risk management, no previous known study in Veterans or non-Veterans has followed rates of BP control initiation or maintenance in this way. Based on these results, the first year of hypertension care appears to be a crucial time for all patients. Among post-9/11 Veterans receiving VA care, less than half of men and 2 of 3 women showed BP control in the year after hypertension onset, with only slight improvement after 5 years of care. Women and Hispanic patients were consistently more likely to achieve BP control, whereas men and NH Black patients warrant greater attention for early hypertension management. Importantly, social deprivation did not significantly alter these associations so the VA system may protect against some of the social inequity observed more broadly. Improving hypertension identification, and expeditious, initial BP control should be prioritized.

Perspectives **COMPETENCY IN MEDICAL KNOWLEDGE:** Hypertension is common among post-9/11 Veterans, yet blood pressure control remains suboptimal despite access to VA care. Within 1 year, fewer than half of men and about two-thirds of women achieved BP control, with minimal improvement over 5 years. Women and Hispanic Veterans showed consistently better control, whereas men and Black non-Hispanic Veterans had persistently lower rates. These disparities persisted even after accounting for social deprivation, emphasizing the need for clinicians to prioritize early hypertension management, particularly in groups who are underserved and high-risk for hypertension.

**TRANSLATIONAL OUTLOOK:** Uniform access to care in the VA system did not eliminate demographic differences in BP control, suggesting that additional factors—for example, adherence, health literacy, and health care engagement—likely play a role. Future research should examine how individual components of social deprivation affect hypertension outcomes and explore tailored interventions that address sex, race, and ethnicity. Strategies such as early pharmacologic intensification, home BP monitoring, and targeted patient education may help improve outcomes in general and particularly for high-risk subgroups.

## Funding support and author disclosures

Dr Gaffey was supported by a 10.13039/100000050National Institutes of Health/National Heart, Lung, and Blood Institute grant (K23HL168233). Dr Chang was supported by an 10.13039/100000968American Heart Association Predoctoral Fellowship (#23PRE1018200/2023-2024). Dr Dhruvawas supported by a grant from the Veterans Affairs Health Services Research & Development (1IK2HX003357). Dr Spatz receives grant funding from the 10.13039/100000030Centers for Disease Control and Prevention (20042801-Sub01), the 10.13039/100000050National Heart, Lung, and Blood Institute (R01HL151240), and the 10.13039/100006093Patient-Centered Outcomes Research Institute (HM-2022C2-28354). Dr Dhruva reported funding from Arnold Ventures, serving on the Institute for Clinical and Economic Review California Technology Assessment Forum and Medicare Evidence Development & Coverage Advisory Committee. The views and opinions of authors expressed herein do not necessarily state or reflect those of the United States Government. All other authors have reported that they have no relationships relevant to the contents of this paper to disclose.
