# Supplementary material for: Blood Pressure Control and Maintenance in U.S. Veterans: Roles of Sex, Race, Ethnicity, and Deprivation
Source: JACC Adv. 2025 Oct 24;4(11):102267. doi: 10.1016/j.jacadv.2025.102267 (PMC12595360; doi:10.1016/j.jacadv.2025.102267)
Supplement: Supplemental Material [file mmc1.docx]

**Supplemental Materials**

**Supplemental Methods**

Qualifying BP measurements included those within biologically plausible ranges (systolic 70-240 mmHg; diastolic 35-140 mmHg^1^). To address multiple BP measurements in a 5-minute period: a) if measurements were identical (i.e., duplicate systolic or diastolic values), one set was deleted; b) if there were 2 unique measurements the means of each systolic and diastolic measurements were used to represent a single BP; c) if there were >2 unique measurements, the first BP measurement was dropped and the mean of the remaining values was used.^2^

References

1. Burg MM, Brandt C, Buta E, et al. Risk for incident hypertension associated with PTSD in military veterans, and the effect of PTSD treatment. Psychosom Med. 2017;79:181. doi:10.1097/PSY.0000000000000376

2. Lu Y, Linderman GC, Mahajan S, et al. Quantifying blood pressure visit-to-visit variability in the real-world setting: A retrospective cohort study. Circ Cardiovasc Qual Outcomes. 2023;16:e009258. doi:10.1161/CIRCOUTCOMES.122.009258

**STROBE Statement**

|  | Item No | Recommendation | Page No |
| --- | --- | --- | --- |
| **Title and abstract** | 1 | (*a*) Indicate the study’s design with a commonly used term in the title or the abstract | 3 |
|  |  | (*b*) Provide in the abstract an informative and balanced summary of what was done and what was found | 3 |
| Introduction | | | |
| Background/rationale | 2 | Explain the scientific background and rationale for the investigation being reported | 6-7 |
| Objectives | 3 | State specific objectives, including any prespecified hypotheses | 7 |
| Methods | | | |
| Study design | 4 | Present key elements of study design early in the paper | 7-10 |
| Setting | 5 | Describe the setting, locations, and relevant dates, including periods of recruitment, exposure, follow-up, and data collection | 7-10 |
| Participants | 6 | (*a*) Give the eligibility criteria, and the sources and methods of selection of participants. Describe methods of follow-up | 7-8 |
|  |  | (*b*) For matched studies, give matching criteria and number of exposed and unexposed | N/A |
| Variables | 7 | Clearly define all outcomes, exposures, predictors, potential confounders, and effect modifiers. Give diagnostic criteria, if applicable | 8-9 |
| Data sources/ measurement | 8* | For each variable of interest, give sources of data and details of methods of assessment (measurement). Describe comparability of assessment methods if there is more than one group | 8-10 |
| Bias | 9 | Describe any efforts to address potential sources of bias | N/A |
| Study size | 10 | Explain how the study size was arrived at | 10-11 |
| Quantitative variables | 11 | Explain how quantitative variables were handled in the analyses. If applicable, describe which groupings were chosen and why | 8-10 |
| Statistical methods | 12 | (*a*) Describe all statistical methods, including those used to control for confounding | 10 |
|  |  | (*b*) Describe any methods used to examine subgroups and interactions |  |
|  |  | (*c*) Explain how missing data were addressed |  |
|  |  | (*d*) If applicable, explain how loss to follow-up was addressed |  |
|  |  | (*e*) Describe any sensitivity analyses |  |
| Results | | |  |
| Participants | 13* | (a) Report numbers of individuals at each stage of study—eg numbers potentially eligible, examined for eligibility, confirmed eligible, included in the study, completing follow-up, and analysed | 10-11 |
|  |  | (b) Give reasons for non-participation at each stage |  |
|  |  | (c) Consider use of a flow diagram |  |
| Descriptive data | 14* | (a) Give characteristics of study participants (eg demographic, clinical, social) and information on exposures and potential confounders | 10-11 |
|  |  | (b) Indicate number of participants with missing data for each variable of interest |  |
|  |  | (c) Summarise follow-up time (eg, average and total amount) |  |
| Outcome data | 15* | Report numbers of outcome events or summary measures over time | 10-13 |

| Main results | 16 | (*a*) Give unadjusted estimates and, if applicable, confounder-adjusted estimates and their precision (eg, 95% confidence interval). Make clear which confounders were adjusted for and why they were included | 10-13 |
| --- | --- | --- | --- |
|  |  | (*b*) Report category boundaries when continuous variables were categorized |  |
|  |  | (*c*) If relevant, consider translating estimates of relative risk into absolute risk for a meaningful time period |  |
| Other analyses | 17 | Report other analyses done—eg analyses of subgroups and interactions, and sensitivity analyses | 12-13 |
| Discussion | | | |
| Key results | 18 | Summarise key results with reference to study objectives | 13 |
| Limitations | 19 | Discuss limitations of the study, taking into account sources of potential bias or imprecision. Discuss both direction and magnitude of any potential bias | 17-18 |
| Interpretation | 20 | Give a cautious overall interpretation of results considering objectives, limitations, multiplicity of analyses, results from similar studies, and other relevant evidence | 13-18 |
| Generalisability | 21 | Discuss the generalisability (external validity) of the study results | 13-18 |
| Other information | | | |
| Funding | 22 | Give the source of funding and the role of the funders for the present study and, if applicable, for the original study on which the present article is based | 1-2 |

**Supplemental Tables**

**Table S1.** Patients who met criteria for hypertension, by sex.

|  | Total  (n=398,732) | Men  (n=358,700) | Women  (*n*=40,032) |
| --- | --- | --- | --- |
| BP only | 101678 (25.5) | 94113 (26.2) | 7565 (18.9) |
| Diagnosis only | 11220 (2.8) | 10463 (2.9) | 757 (1.9) |
| AHM only | 42410 (10.6) | 33118 (9.2) | 9292 (23.2) |
| Diagnosis and BP | 12662 (3.2) | 11905 (3.3) | 757 (1.9) |
| AHM and BP | 50553 (12.7) | 44787 (12.5) | 5766 (14.4) |
| AHM, diagnosis | 34503 (8.7) | 31353 (8.7) | 3150 (7.9) |
| AHM, diagnosis, and BP | 145706 (36.5) | 132961 (37.1) | 12745 (31.8) |

Abbreviations: AHM, antihypertensive medication; BP, blood pressure; HTN, hypertension

Notes. Hypertension was defined if an individual had a diagnosis code, an antihypertensive medication fill, and/or BP readings of 140/90 mmHg.

**Table S2.** Patients who met criteria for hypertension, by sex, race, and ethnicity (n=398,732).

|  | Men (*n*=358,700) | | | | | | Women (*n*=40,032) | | | | | | |
| --- | --- | --- | --- | --- | --- | --- | --- | --- | --- | --- | --- | --- | --- |
|  | **Total** | **NH White**  ***n*=234381** | **NH Black *n*=67448** | **Hispanic *n*=37203** | **NH Asian *n*=8646** | **NH Other *n*=11022** | **Total** | **NH White *n*=17964** | **NH Black *n*=15800** | **Hispanic *n*=3793** | **NH Asian *n*=896** | **NH Other *n*=1579** |  |
| BP only | 94113 (26.2) | 65836 (28.1) | 12788 (19.0) | 10804 (29.0) | 1865 (21.6) | 2820 (25.6) | 7565 (18.9) | 3986 (22.2) | 2340 (14.8) | 771 (20.3) | 159 (17.8) | 309 (19.6) |  |
| Diagnosis only | 10463 (2.9) | 6853 (2.9) | 1995 (3.0) | 959  (2.6) | 362 (4.2) | 294 (2.7) | 757 (1.9) | 289 (1.5) | 361 (2.3) | 68  (1.8) | 18 (2.0) | 21 (1.3) |  |
| AHM only | 33118 (9.2) | 23049 (9.8) | 4252 (6.3) | 3950 (10.6) | 869 (10.1) | 998 (9.1) | 9292 (23.2) | 4924 (27.4) | 2568 (16.3) | 1205 (31.8) | 200 (22.3) | 395 (25.0) |  |
| Diagnosis and BP | 11905 (3.3) | 7890 (3.4) | 2244 (3.3) | 1148 (3.1) | 265 (3.1) | 358 (3.3) | 757 (1.9) | 290 (1.6) | 355 (2.3) | 58  (1.5) | 20 (2.3) | 34 (2.2) |  |
| AHM and BP | 44787 (12.5) | 31749 (13.6) | 5879 (8.7) | 4838 (13.0) | 909 (10.5) | 1412 (12.8) | 5766 (14.4) | 3032 (16.9) | 1815 (11.5) | 582 (15.3) | 103 (11.5) | 234 (14.8) |  |
| AHM, diagnosis | 31353 (8.7) | 19142 (8.2) | 7018 (10.4) | 3096 (8.3) | 1129 (13.1) | 968 (8.8) | 3150 (7.9) | 1131 (6.3) | 1567 (9.9) | 222  (5.9) | 104 (11.6) | 126 (8.0) |  |
| AHM, diagnosis, BP | 132961 (37.1) | 79862 (34.1) | 33272 (49.3) | 12408 (33.4) | 3247 (37.6) | 4172 (37.9) | 12745 (31.8) | 4312 (24.0) | 6794 (43.0) | 87  (23.4) | 292 (32.6) | 460 (29.1) |  |

Abbreviations: AHM, antihypertensive medication; BP, blood pressure; NH, non-Hispanic

Notes. Hypertension was defined if an individual had a diagnosis code, an antihypertensive medication fill, and/or BP readings of 140/90 mmHg.

**Table S3.** Demographic, behavioral, and clinical characteristics, presented by sex, race, and ethnicity (n=398,732).

|  | **Men (*n*=358,700)** | | | | | **Women (*n*=40,032)** | | | | |
| --- | --- | --- | --- | --- | --- | --- | --- | --- | --- | --- |
|  | **NH White**  ***n*=234381** | **NH Black *n*=67448** | **Hispanic *n*=37203** | **NH Asian *n*=8646** | **NH Other *n*=11022** | **NH White *n*=17964** | **NH Black *n*=15800** | **Hispanic *n*=3793** | **NH Asian *n*=896** | **NH Other *n*=1579** |
| **Demographics** | |  |  |  |  |  |  |  |  |  |
| **Age, years** | 36.6 (30.3,44.6) | 41.4 (32.9,48.7) | 35.7 (29.6,43.8) | 40.8 (33.0,47.2) | 35.9 (29.3,45.1) | 37.4 (31.1,45.3) | 40.1 (33.3,47.6) | 38.9 (30.3,43.3) | 40.2 (32.8,47.1) | 36.6 (30.1,45.6) |
| **Married** | 131069 (56.4) | 37352 (55.8) | 20777 (56.2) | 5401  (62.9) | 6158  (56.3) | 7223  (40.5) | 5318  (33.9) | 1481  (39.4) | 424  (47.8) | 620  (39.7) |
| **>HS**  **Education** | 25,596 (23.0) | 7,514 (19.6) | 3,186 (18.4) | 1,188 (29.2) | 1,264  (20.2) | 2,649  (29.5) | 2,593 (29.3) | 377  (21.7) | 162  (39.3) | 198  (24.1) |
| **Rural**  **Residence** | 83227 (35.5) | 10846 (16.1) | 5097  (13.7) | 667  (7.7) | 2930  (26.6) | 5553  (30.9) | 2026  (12.8) | 421  (11.1) | 77  (8.6) | 326  (20.7) |
| **Other health insurance** | 99822 (42.7) | 27884 (41.4) | 14244 (38.3) | 4060  (47.1) | 4369  (39.7) | 7603  (42.4) | 6632  (42.0) | 1410  (37.2) | 401  (44.8) | 605  (38.3) |
| **SDI** |  |  |  |  |  |  |  |  |  |  |
| **Q1** | 71592 (30.6) | 10618 (15.7) | 6286  (16.0) | 1987  (23.0) | 2416  (21.9) | 5321  (29.6) | 2426  (15.4) | 684  (18.0) | 217  (24.2) | 325  (20.6) |
| **Q2** | 62513 (26.7) | 13893 (20.6) | 7483  (20.1) | 2360  (27.3) | 2789  (25.3) | 4809  (26.8) | 3424  (21.7) | 828  (21.8) | 252  (28.1) | 404  (25.6) |
| **Q3** | 58439 (24.9) | 17146 (25.4) | 8054  (21.7) | 1633  (18.9) | 2643  (24.0) | 4466  (24.9) | 4255  (26.9) | 817  (21.5) | 180  (20.1) | 362  (22.9) |
| **Q4** | 41837 (17.9) | 25791 (38.2) | 15380 (41.3) | 2666  (30.8) | 3174  (28.8) | 3368  (18.8) | 5695  (36.0) | 1464  (38.6) | 247  (27.6) | 488  (30.9) |
| **Behavioral factors** | |  |  |  |  |  |  |  |  |  |
| **Current smokers** | 112083 (48.2) | 22093 (32.9) | 11729 (31.7) | 2531  (29.5) | 4724  (43.2) | 6180  (34.6) | 3104  (19.7) | 761  (20.1) | 164  (18.4) | 482  (30.7) |
| **Obese** | 122101 (53.5) | 39605 (59.8) | 22602 (62.0) | 3338  (39.9) | 6333  (58.9) | 7737  (44.0) | 8692  (55.8) | 1796  (48.5) | 252  (29.5) | 758  (49.3) |
| **Alcohol or drug use disorder** | 70988 (30.3) | 18096 (26.8) | 11415 (30.7) | 1387  (16.0) | 3374  (30.6) | 3751  (20.9) | 2663  (16.9) | 695  (18.3) | 80  (8.9) | 328  (20.8) |
| **Clinical factors** |  |  |  |  |  |  |  |  |  |  |
| **MST** | 5162  (2.2) | 1474  (2.2) | 861  (2.3) | 187  (2.2) | 283  (2.6) | 6312  (35.6) | 4742  (30.4) | 1438  (38.4) | 247  (28.2) | 608  (39.1) |
| **PTSD** | 132026 (56.3) | 39607 (58.7) | 23696 (63.7) | 3864  (44.7) | 6755  (61.3) | 9720  (54.1) | 9043  (57.2) | 2341  (61.7) | 382  (42.6) | 909  (57.6) |
| **MDD** | 92597 (39.5) | 27322 (40.5) | 16297 (43.8) | 3119  (36.1) | 4642  (42.1) | 9892  (55.1) | 9337  (59.1) | 2317  (61.1) | 387  (43.2) | 901  (57.1) |
| **GAD** | 27664 (11.8) | 4736 (7.0) | 3821 (10.3) | 708  (8.2) | 1033  (9.4) | 3770  (21.0) | 2352  (14.9) | 695  (18.3) | 118  (13.2) | 246  (15.6) |
| **Diabetes** | 27450 (11.7) | 15213 (22.6) | 6535 (17.6) | 1964  (22.7) | 2045  (18.6) | 1370  (7.6) | 2666  (16.9) | 449  (11.8) | 155  (17.3) | 225  (14.3) |
| **Lipid disorders** | 114747 (49.0) | 34143 (50.6) | 19054 (51.2) | 4940  (57.1) | 5539  (50.3) | 6411  (35.7) | 5570  (35.3) | 1312  (34.6) | 386  (43.1) | 538  (34.1) |
| **OSA** | 77884 (33.2) | 29667 (44.0) | 15869 (42.7) | 3781  (43.7) | 4313  (39.1) | 3411  (19.0) | 4357  (27.6) | 918  (24.2) | 206  (23.0) | 363  (23.0) |
| **Comorbidities** |  |  |  |  |  |  |  |  |  |  |
| **0** | 96626 (41.2) | 22353 (33.1) | 13580 (36.5) | 3094  (35.8) | 4218  (38.3) | 5984  (33.3) | 4578  (29.0) | 1205  (31.8) | 290  (32.4) | 539  (34.1) |
| **1** | 83236 (35.5) | 23777 (35.3) | 13736 (36.9) | 3116  (36.0) | 3888  (35.3) | 7282  (40.5) | 6450  (40.8) | 1558  (41.1) | 354  (39.5) | 608  (38.5) |
| **2** | 29866 (12.7) | 10007 (14.8) | 5370  (14.4) | 1356  (15.7) | 1572  (14.3) | 2840  (15.8) | 2780  (17.6) | 636  (16.8) | 134  (15.0) | 258  (16.3) |
| **≥3** | 24653 (10.5) | 11311 (16.8) | 4517  (12.1) | 1080  (12.5) | 1344  (12.2) | 1858  (10.3) | 1992  (12.6) | 394  (10.3) | 118  (13.2) | 174  (11.0) |
| **Primary care visits** | 5  (3,8) | 5  (3,8) | 5  (3,8) | 5  (3,8) | 5  (3,8) | 7  (4,10) | 6  (4,10) | 7  (4,11) | 7  (4,11) | 7  (4,11) |
| **Follow-up** | 5.4 (1.9,9.6) | 6.1 (2.4,10.4) | 5.2 (1.9,9.1) | 5.2 (1.9,9.1) | 5.2 (1.8,9.1) | 4.8 (1.7,8.7) | 5.6 (2.2,9.7) | 4.6 (1.7,7.9) | 4.2 (1.4,7.7) | 4.3 (1.7,8.0) |
| **Baseline BP category** |  |  |  |  |  |  |  |  |  |  |
| **≥160/100 mmHg** | 15013 (6.4) | 6176  (9.2) | 2139  (5.8) | 702  (8.1) | 857  (7.8) | 570  (3.2) | 796  (5.0) | 102  (2.7) | 45  (5.0) | 70  (4.4) |
| **140-159/90-99 mmHg** | 65945 (28.1) | 19460 (28.9) | 10182 (27.4) | 2394  (27.7) | 3259  (29.6) | 2666  (14.8) | 2879  (18.2) | 511  (13.5) | 149  (16.6) | 304  (193) |
| **130-139/80-89 mmHg** | 89416 (38.2) | 24177 (35.9) | 14104 (37.9) | 3235  (37.4) | 4075  (37.0) | 5872  (32.7) | 5364  (34.0) | 1168  (30.8) | 307  (34.3) | 507  (32.1) |
| **120-129/<80 mmHg** | 30995 (13.2) | 8584  (12.7) | 5282  (14.2) | 1017  (11.8) | 1352  (12.3) | 2593  (14.4) | 2142  (13.6) | 520  (13.7) | 101  (11.3) | 201  (12.7) |
| **<120/80 mmHg** | 33012 (14.1) | 9051  (13.4) | 5496  (14.8) | 1298  (15.0) | 1479  (13.4) | 6263  (34.9) | 4619  (29.2) | 1492  (39.3) | 294  (32.8) | 497  (31.5) |
| **First AHM category prescribed** | 37670 (16.1) | 9855  (14.6) | 6273  (16.9) | 1633  (18.9) | 1997  (18.1) | 1446  (8.1) | 1324  (8.4) | 309  (8.2) | 107  (11.9) | 158  (10.0) |
| **ACE inhibitor** | 7620 (3.3) | 2557  (3.8) | 1342  (3.6) | 585  (6.8) | 480  (4.4) | 383  (2.1) | 394  (2.5) | 76  (2.0) | 46  (5.1) | 43  (2.7) |
| **ARB** | 45777 (19.5) | 8373  (12.4) | 6843  (18.4) | 1497  (17.3) | 2023  (18.4) | 5732  (31.9) | 3347  (21.2) | 1275  (33.6) | 208  (23.2) | 463  (29.3) |
| **Beta blocker** | 18076 (7.7) | 11182 (16.6) | 3093  (8.3) | 897  (10.4) | 960  (8.7) | 1368  (7.6) | 2265  (14.3) | 300  (7.9) | 99  (11.1) | 144  (9.1) |
| **Calcium channel blocker** | 22613 (9.7) | 11156 (16.5) | 3653  (9.8) | 927  (10.7) | 1133  (10.3) | 3028  (16.9) | 3854  (24.4) | 679  (17.9) | 169  (18.9) | 286  (18.1) |
| **Diuretic** | 11051 (4.7) | 5871  (8.7) | 1761  (4.7) | 442  (5.1) | 555  (5.0) | 586  (3.3) | 1162  (7.4) | 125  (3.3) | 37  (4.1) | 59  (3.7) |
| **Diuretic combo with other class** | 339  (0.1) | 313  (0.5) | 66  (0.2) | 18  (0.2) | 23  (0.2) | 11  (0.1) | 45  (0.3) | 3  (0.1) | 2  (0.2) | 3  (0.2) |
| **Other class combo (no diuretic)** | 10656 (4.6) | 1114  (1.7) | 1261  (3.4) | 155  (1.8) | 379  (3.4) | 845  (4.7) | 353  (2.2) | 129  (3.4) | 31  (3.5) | 59  (3.7) |
| **Other medications** | 37670 (16.1) | 9855  (14.6) | 6273  (16.9) | 1633  (18.9) | 1997  (18.1) | 1446  (8.1) | 1324  (8.4) | 309  (8.2) | 107  (11.9) | 158  (10.0) |

Abbreviations: GAD, generalized anxiety disorder; IQR, interquartile range; MDD, major depressive disorder; MST, military sexual trauma; NH, non-Hispanic; OSA, obstructive sleep apnea; PTSD, posttraumatic stress disorder; SDI, Social Deprivation Index

Notes. Data are presented as N (%). All other data are presented as median (Q1, Q3). P-value tests differences across all sex, race, and ethnicity groups using chi-square test for categorical variables, Kruskal-Wallis test for continuous non-normally distributed variables. If an individual was in 2 distinct categories based on systolic and diastolic BP, they were placed in the higher BP category. SDI Quartile 1=least disadvantaged and Quartile 4=most disadvantaged

**Table S4.** Blood pressure control at 1-5 years after hypertension, by demographic, behavioral, and clinical factors.

|  |  | | | **Year 1** | | **Year 2** | | **Year 5** | |
| --- | --- | --- | --- | --- | --- | --- | --- | --- | --- |
|  |  | | **Men** | | **Women** | **Men** | **Women** | **Men** | **Women** |
| **Total** |  | | 149,067 (43.1) | | 23,230 (60.4) | 144484 (59.0) | 20,447 (69.7) | 100,198 (58.6) | 13,054 (66.5) |
| **Demographics** | |  | | |  |  |  |  |  |
| **Age, years** | | 83,314 (40.9) | | | 81,469 (58.8) | 56,348 (58.5) | 13,634 (63.3) | 11,789 (72.2) | 7,548 (69.5) |
| **<40** | | 65,753 (46.1) | | | 63,015 (59.4) | 43,850 (58.7) | 9,596 (56.6) | 8,658 (66.7) | 5,506 (62.7) |
| **≥40** | |  | | |  |  |  |  |  |
| **Race and ethnicity** | | 96,587 (42.7) | | | 94,557 (59.5) | 65,210 (59.5) | 10,696 (62.0) | 9,467 (72.5) | 5,997 (70.3) |
| **NH White** | | 27,912 (42.9) | | | 26,398 (55.6) | 18,918 (53.7) | 8,802 (57.7) | 7,770 (65.9) | 5,119 (61.7) |
| **NH Black** | | 16,121 (45.0) | | | 15,655 (61.9) | 10,544 (61.1) | 2,364 (65.2) | 2,017 (73.9) | 1,250 (71.2) |
| **Hispanic** | | 3,946 (47.7) | | | 3,622 (61.8) | 2,513 (62.5) | 496 (57.7) | 420 (68.3) | 241 (63.4) |
| **NH Asian** | | 4,501 (42.4) | | | 4,252 (58.1) | 3,013 (59.6) | 872 (57.8) | 773 (68.5) | 447 (65.4) |
| **NH Other** | |  | | |  |  |  |  |  |
| **Married** | | 87,180 (45.1) | | | 84,693 (60.0) | 59,693 (59.7) | 8,832 (61.1) | 7,769 (70.7) | 4,291 (68.2) |
| **Rural** | | 42,072 (42.4) | | | 42,365 (58.9) | 30,721 (58.9) | 4,827 (59.8) | 4,384 (69.7) | 2,878 (66.7) |
| **Additional health insurance** | | 65,882 (45.4) | | | 65,676 (60.4) | 48,648 (60.2) | 9,675 (60.4) | 8,839 (69.5) | 6,018 (66.5) |
| **Social deprivation** | |  | | |  |  |  |  |  |
| **Quartile 1 (least disadvantaged)** | | 38,605 (43.1) | | | 37,665 (59.5) | 25,517 (58.8) | 5,245 (60.8) | 4,570 (70.4) | 2,818 (67.3) |
| **Quartile 2** | | 37,371 (43.5) | | | 36,134 (59.4) | 24,906 (58.9) | 5,685 (61.1) | 4,946 (69.4) | 3,192 (67.6) |
| **Quartile 3** | | 36,841 (43.4) | | | 35,848 (59.3) | 25,111 (59.0) | 5,848 (60.3) | 5,192 (70.2) | 3,278 (65.6) |
| **Quartile 4 (most disadvantaged)** | | 36,250 (42.2) | | | 34,837 (57.9) | 24,664 (57.5) | 6,452 (59.5) | 5,739 (68.8) | 3,766 (65.7) |
| **Behavioral/lifestyle factors** | |  | | |  |  |  |  |  |
| **Current smoker** | | 29,072 (44.1) | | | 28,581 (60.2) | 19,653 (60.0) | 3,507 (61.4) | 3,042 (70.7) | 1,913 (68.4) |
| **Obese** | | 77,507 (41.4) | | | 76,781 (56.3) | 54,962 (55.9) | 10,729 (57.7) | 9,588 (66.1) | 6,475 (63.8) |
| **Alcohol or drug use disorder** | | 43,403 (42.8) | | | 41,967 (56.8) | 30,555 (56.4) | 4,324 (60.2) | 3,842 (68.8) | 2,512 (64.9) |
| **Clinical factors** | |  | | |  |  |  |  |  |
| **PTSD** | | 3,552 (46.3) | | | 3,321 (60.0) | 2,273 (59.8) | 8,061 (62.9) | 6,936 (70.7) | 4,377 (67.1) |
| **MDD** | | 89,672 (45.2) | | | 87,191 (59.2) | 62,757 (58.6) | 13,627 (63.4) | 11,961 (71.0) | 7,835 (67.3) |
| **GAD** | | 63,441 (45.7) | | | 61,739 (59.0) | 44,753 (58.2) | 13,922 (63.4) | 12,240 (70.5) | 8,070 (67.1) |
| **Diabetes** | | 17,046 (46.7) | | | 16,474 (59.7) | 11,794 (58.9) | 4,496 (65.2) | 3,938 (72.8) | 2,535 (68.5) |
| **Dyslipidemia** | | 24,244 (47.4) | | | 23,455 (56.9) | 18,874 (56.8) | 2,702 (57.5) | 2,490 (63.1) | 1,904 (61.4) |
| **OSA** | | 78,317 (45.5) | | | 78758 (58.8) | 59,512 (58.4) | 8,030 (58.4) | 7,546 (67.1) | 5,368 (64.0) |
| **Military sexual trauma** | | 3552 (46.3) | | | 8061 (62.9) | 3321 (60.0) | 6936 (70.7) | 2273 (59.8) | 4377 (67.1) |
| **Number of comorbidities** | |  | | |  |  |  |  |  |
| **0** | | 51,857 (38.6) | | | 6812 (56.9) | 47790 (59.3) | 5645 (70.8) | 27810 (59.2) | 2,927 (67.9) |
| **1** | | 55,172 (44.7) | | | 9765 (62.3) | 55063 (59.8) | 8735 (71.2) | 38775 (59.1) | 5,634 (67.5) |
| **2** | | 21,915 (51.1) | | | 4025 (62.5) | 21916 (58.5) | 3661 (68.9) | 17109 (58.0) | 2,584 (65.4) |
| **≥3** | | 20,123 (46.9) | | | 2,628 (57.9) | 20,715 (48.3) | 2,406 (53.0) | 16,504 (38.4) | 1,909 (42.1) |
| **Number of primary care visits** | | 5 (3,9) | | | 7 (4,11) | 6 (3,9) | 7 (4,11) | 6 (3,9) | 8 (4,12) |
| **Follow-up period** | | 5.6 (2.2,9.7) | | | 5.1 (2.0,8.9) | 6.4 (3.3,10.2) | 5.9 (3.0,9.5) | 8.5 (5.8,11.8) | 7.9 (5.6,11.1) |
| **Baseline BP category†** | |  | | |  |  |  |  |  |
| **≥160/100 mmHg** | | 5241 (21.6) | | | 442 (28.7) | 5,904 (36.6) | 454 (40.3) | 4,891 (40.6) | 374 (44.6) |
| **140-159/90-99 mmHg** | | 30789 (31.2) | | | 2,523 (39.7) | 33,436 (50.5) | 2,523 (54.5) | 25,026 (51.8) | 1,776 (53.9) |
| **130-139/80-89 mmHg** | | 60,009 (46.2) | | | 7,370 (57.7) | 56,912 (60.5) | 6,484 (66.5) | 38,905 (59.7) | 4,124 (62.6) |
| **120-129/<80 mmHg** | | 23,849 (52.5) | | | 3,476 (65.2) | 22,127 (67.4) | 2,993 (73.1) | 14,725 (65.7) | 1,971 (71.2) |
| **<120/80 mmHg** | | 29,179 (60.8) | | | 9,419 (75.4) | 26,105 (73.5) | 7,993 (82.2) | 16,651 (71.7) | 4,809 (78.2) |
| **First AHM prescribed** | |  | | |  |  |  |  |  |
| **ACE inhibitor** | | 23,784 (43.3) | | | 1,649 (51.4) | 22,854 (54.5) | 1,504 (57.6) | 17,072 (54.4) | 1,049 (56.2) |
| **ARB** | | 4,936 (41.0) | | | 454 (49.7) | 4,677 (52.9) | 402 (57.5) | 2,969 (50.8) | 250 (55.9) |
| **Beta blocker** | | 35,552 (58.8) | | | 7,861 (75.8) | 30,913 (66.6) | 6,493 (79.1) | 21,182 (64.8) | 4,039 (75.2) |
| **Calcium channel blocker** | | 12,759 (38.8) | | | 2,121 (52.7) | 12,332 (50.3) | 1,965 (63.0) | 8,863 (49.6) | 1,344 (59.9) |
| **Diuretic** | | 13,986 (36.9) | | | 4,638 (60.6) | 13,994 (48.8) | 4,042 (66.4) | 11,017 (49.6) | 2,722 (61.6) |
| **Diuretic combo with other class** | | 7,654 (40.7) | | | 1,065 (56.0) | 7,501 (52.1) | 908 (60.8) | 5,896 (52.4) | 654 (56.4) |
| **Other class combo (no diuretic)** | | 319 (43.8) | | | 34 (55.7) | 278 (54.2) | 34 (70.8) | 204 (57.8) | 18 (60.0) |
| **Other medications** | | 7,525 (59.5) | | | 988 (73.9) | 6,420 (66.5) | 820 (77.8) | 4,446 (65.8) | 491 (73.4) |

Abbreviations: ACE inhibitor, angiotensin-converting enzyme inhibitor; AHM, antihypertensive medication; ARB, angiotensin receptor blocker; BP, blood pressure; BMI, body mass index; EHR, electronic health record; GAD, generalized anxiety disorder; MDD, major depressive disorder; MST, military sexual trauma; NH, non-Hispanic; OSA, obstructive sleep apnea; PTSD, posttraumatic stress disorder; SDI, Social Deprivation Index

Data are presented as N (%) or median (Q1, Q3). Other medications were vasodilators, direct renin inhibitors, and alpha central 2 antagonists.

**Table S5.** Multivariable models for the effect of sex on BP control odds at year 1, by race and ethnicity.

|  | NH White | | NH Black | | Hispanic | |  | NH Asian | | NH Other | |
| --- | --- | --- | --- | --- | --- | --- | --- | --- | --- | --- | --- |
|  | **OR**  **(95% CI)** | ***P*** | **OR**  **(95% CI)** | ***P*** | **OR**  **(95% CI)** | ***P*** |  | **OR**  **(95% CI)** | ***P*** | **OR**  **(95% CI)** | ***P*** |
| Women | 1.96  (1.89-2.04) | <0.001 | 1.74  (1.67-1.81) | <0.001 | 2.07  (1.90-2.26) | <0.001 |  | 1.26  (1.07-1.48) | 0.007 | 1.79  (1.57-2.04) | <0.001 |
| Age <40 years  (vs 40+ years) | 1.10  (1.08-1.12) | <0.001 | 1.07  (1.03-1.10) | <0.001 | 1.09  (1.04-1.14) | <0.001 |  | 1.12  (1.01-1.23) | 0.032 | 1.08  (1.00-1.18) | 0.063 |
| Not married  (vs married) | 0.89  (0.87-0.90) | <0.001 | 0.88  (0.85-0.90) | <0.001 | 0.92  (0.88-0.96) | <0.001 |  | 0.92  (0.83-1.01) | 0.074 | 0.84  (0.77-0.91) | <0.001 |
| Current smoker  (vs never) | 0.90  (0.88-0.91) | <0.001 | 0.83  (0.80-0.86) | <0.001 | 0.92  (0.87-0.97) | 0.001 |  | 0.89  (0.80-0.99) | 0.036 | 0.92  (0.84-1.00) | 0.062 |
| Past smoker  (vs never) | 0.98  (0.96-1.01) | 0.133 | 0.99  (0.94-1.04) | 0.606 | 0.96  (0.91-1.02) | 0.165 |  | 1.00  (0.90-1.11) | 0.957 | 0.99  (0.89-1.10) | 0.818 |
| Overweight  (vs normal/under) | 0.77  (0.75-0.80) | <0.001 | 0.82  (0.78-0.87) | <0.001 | 0.74  (0.68-0.80) | <0.001 |  | 0.74  (0.65-0.84) | <0.001 | 0.83  (0.73-0.96) | 0.009 |
| Obese  (vs normal/under) | 0.63  (0.61-0.64) | <0.001 | 0.69  (0.65-0.72) | <0.001 | 0.59  (0.54-0.64) | <0.001 |  | 0.60  (0.52-0.69) | <0.001 | 0.62  (0.55-0.71) | <0.001 |
| Substance use disorder (vs never) | 0.93  (0.91-0.95) | <0.001 | 0.96  (0.92-1.00) | 0.028 | 0.92  (0.88-0.97) | 0.001 |  | 0.85  (0.74-0.96) | 0.011 | 0.90  (0.82-0.99) | 0.022 |
| Number of comorbidities | 1.03  (1.02-1.03) | <0.001 | 1.02  (1.02-1.03) | <0.001 | 1.03  (1.03-1.04) | <0.001 |  | 1.02  (1.01-1.03) | <0.001 | 1.03  (1.02-1.04) | <0.001 |
| PTSD ever  (vs never) | 1.20  (1.18-1.22) | <0.001 | 1.17  (1.14-1.21) | <0.001 | 1.22  (1.17-1.28) | <0.001 |  | 1.20  (1.10-1.32) | 0.001 | 1.13  (1.04-1.23) | 0.003 |
| OSA ever  (vs never) | 1.04  (1.04-1.05) | <0.001 | 1.01  (1.00-1.02) | 0.150 | 1.02  (1.00-1.04) | 0.016 |  | 1.08  (1.04-1.11) | <0.001 | 1.02  (0.99-1.05) | 0.270 |
| Diabetes  (vs none) | 1.09  (1.07-1.11) | 0.005 | 1.05  (1.02-1.09) | 0.004 | 1.16 (1.11-1.22) | 0.268 |  | 0.98  (0.90-1.08) | 0.079 | 1.02  (0.94-1.11) | 0.188 |
| Dyslipidemia  (vs none) | 1.04  (1.01-1.08) | <0.001 | 1.08  (1.03-1.12) | 0.005 | 1.04  (0.97-1.11) | 0.001 |  | 1.12  (0.99-1.27) | 0.037 | 1.08  (0.96-1.21) | 0.030 |
| MST ever  (vs no history) | 1.11  (1.06-1.16) | <0.001 | 1.02  (0.96-1.08) | 0.553 | 1.06  (0.96-1.18) | 0.269 |  | 1.32  (1.05-1.66) | 0.016 | 1.02  (0.86-1.20) | 0.865 |
| Primary care utilization | 1.06  (1.04-1.08) | <0.001 | 1.05  (1.01-1.08) | <0.001 | 1.08  (1.03-1.13) | <0.001 |  | 1.11  (1.01-1.22) | <0.001 | 1.10  (1.01-1.19) | <0.001 |
| Abbreviations: CI, confidence interval; MST, military sexual trauma; NH, non-Hispanic; OR, odds ratio; OSA, obstructive sleep apnea; PTSD, posttraumatic stress disorder;  Notes. Primary care utilization was based on number of visits in the first two years of VA care. Covariates were measured at baseline (demographics, smoking status, BMI, history of military sexual trauma) or by the first diagnosis (substance use, clinical risk factors). | | | | | | | | | | | |

**Table S6.** Multivariable models for the effect of sex on BP control odds at year 2, by race and ethnicity.

|  | NH White | | NH Black | | | Hispanic | | | NH Asian | | | NH Other | | |  |
| --- | --- | --- | --- | --- | --- | --- | --- | --- | --- | --- | --- | --- | --- | --- | --- |
|  | **OR (95% CI)** | ***P*** | | **OR (95% CI)** | ***P*** | | **OR (95% CI)** | ***P*** | | **OR (95% CI)** | ***P*** | | **OR (95% CI)** | ***P*** | |
| Women | 1.63  (1.56-1.70) | <0.001 | | 1.48  (1.41-1.55) | <0.001 | | 1.61  (1.45-1.78) | <0.001 | | 1.21  (0.98-1.49) | 0.071 | | 1.59  (1.35-1.86) | <0.001 | |
| Age <40 years  (vs 40+ years) | 1.00  (0.97-1.02) | 0.658 | | 1.00  (0.96-1.04) | 0.877 | | 0.91  (0.86-0.96) | 0.0008 | | 1.00  (0.88-1.12) | 0.945 | | 0.99  (0.89-1.09) | 0.829 | |
| Not married  (vs married) | 0.90  (0.88-0.92) | <0.001 | | 0.92  (0.90-0.96) | <0.001 | | 0.93  (0.88-0.98) | 0.0061 | | 1.04  (0.92-1.17) | 0.515 | | 0.94  (0.86-1.04) | 0.229 | |
| Past smoker  (vs never) | 1.00  (0.97-1.03) | 0.886 | | 0.97  (0.92-1.03) | 0.302 | | 0.98  (0.92-1.04) | 0.488 | | 0.89  (0.79-1.01) | 0.080 | | 0.99  (0.88-1.12) | 0.901 | |
| Current smoker  (vs never) | 0.91  (0.89-0.93) | <0.001 | | 0.85  (0.82-0.89) | <0.001 | | 0.95  (0.90-1.01) | 0.107 | | 0.77  (0.67-0.88) | 0.001 | | 0.89  (0.80-0.99) | 0.028 | |
| Overweight  (vs normal/under) | 0.76  (0.74-0.79) | <0.001 | | 0.81  (0.76-0.87) | <0.001 | | 0.74  (0.66-0.82) | <0.001 | | 0.74  (0.62-0.87) | <0.001 | | 0.88  (0.74-1.05) | 0.159 | |
| Obese  (vs normal/under) | 0.59  (0.57-0.61) | <0.001 | | 0.68  (0.64-0.72) | <0.001 | | 0.58  (0.52-0.64) | <0.001 | | 0.61  (0.51-0.72) | <0.001 | | 0.66  (0.56-0.78) | <0.001 | |
| Substance use disorder (vs never) | 0.84  (0.82-0.86) | <0.001 | | 0.90  (0.86-0.94) | <0.001 | | 0.86  (0.82-0.92) | <0.001 | | 0.91  (0.78-1.05) | 0.203 | | 0.85  (0.76-0.94) | 0.003 | |
| Number of comorbidities | 1.00  (1.00-1.01) | 0.503 | | 1.00  (1.00-1.00) | 0.371 | | 1.00  (0.95-1.06) | 0.9321 | | 1.00  (0.99-1.01) | 0.300 | | 1.01  (1.00-1.02) | 0.026 | |
| PTSD | 1.07  (1.05-1.10) | <0.001 | | 1.11  (1.07-1.15) | <0.001 | | 0.99  (0.97-1.01) | 0.0086 | | 1.07  (0.95-1.19) | 0.268 | | 1.02  (0.92-1.13) | 0.690 | |
| OSA | 0.98  (0.98-0.99) | <0.001 | | 0.98  (0.97-0.99) | <0.001 | | 1.00  (1.00-1.01) | 0.3559 | | 1.00  (0.96-1.04) | 0.854 | | 0.99  (0.95-1.02) | 0.372 | |
| Diabetes | 0.99  (0.97-1.02) | 0.806 | | 0.98  (0.95-1.02) | <0.001 | | 0.95  (0.88-1.02) | 0.185 | | 0.94  (0.85-1.05) | 0.774 | | 0.90  (0.81-0.99) | 0.456 | |
| Hyperlipidemia | 1.00  (0.96-1.03) | 0.608 | | 0.92  (0.88-0.97) | 0.655 | | 0.99  (0.94-1.05) | 0.831 | | 1.02  (0.88-1.18) | 0.7186 | | 0.95  (0.84-1.08) | 0.604 | |
| MST | 1.10  (1.04-1.16) | <0.001 | | 1.01  (0.94-1.09) | 0.7175 | | 1.00  (0.88-1.13) | 0.9414 | | 0.93  (0.71-1.23) | 0.605 | | 0.86  (0.71-1.05) | 0.134 | |
| Primary care utilization | 0.99  (0.97-1.02) | <0.001 | | 0.99  (0.96-1.03) | 0.759 | | 1.00  (0.98-1.01) | 0.142 | | 1.02  (0.91-1.15) | 0.308 | | 1.03  (0.93-1.13) | 0.059 | |
| Abbreviations: CI, confidence interval; MST, military sexual trauma; NH, non-Hispanic; OR, odds ratio; OSA, obstructive sleep apnea; PTSD, posttraumatic stress disorder;  Notes. Primary care utilization was based on number of visits in the first two years of VA care. Covariates were measured at baseline (demographics, smoking status, BMI, history of military sexual trauma) or by the first diagnosis (substance use, clinical risk factors). | | | | | | | | | | | | | | | |

**Table S7.** Multivariable models for the effect of sex on BP control odds at year 5, by race and ethnicity.

|  | NH White | | NH Black | | Hispanic | | | NH Asian | | NH Other | |  |
| --- | --- | --- | --- | --- | --- | --- | --- | --- | --- | --- | --- | --- |
|  | **OR**  **(95% CI)** | ***P*** | **OR**  **(95% CI)** | ***P*** | | **OR**  **(95% CI)** | ***P*** | **OR**  **(95% CI)** | ***P*** | **OR**  **(95% CI)** | ***P*** | |
| Women | 1.47  (1.39-1.56) | <0.001 | 1.37 (1.30-1.46) | <0.001 | | 1.43  (1.26-1.62) | <0.001 | 0.88  (0.68-1.14) | 0.343 | 1.14  (0.93-1.39) | 0.212 | |
| Age <40 years  (vs 40+ years) | 0.95  (0.92-0.97) | 0.001 | 1.04 (1.00-1.08) | 0.083 | | 0.96  (0.89-1.02) | 0.185 | 0.92  (0.79-1.07) | 0.276 | 0.98  (0.86-1.11) | 0.727 | |
| Not married  (vs married) | 0.89  (0.87-0.92) | <0.001 | 0.88  (0.84-0.92) | <0.001 | | 0.86  (0.81-0.92) | <0.001 | 0.91  (0.79-1.05) | 0.194 | 0.94  (0.83-1.06) | 0.289 | |
| Current smoker  (vs never) | 0.92  (0.89-0.94) | <0.001 | 0.89  (0.85-0.93) | <0.001 | | 0.96  (0.89-1.03) | 0.223 | 0.84  (0.72-0.99) | 0.038 | 0.94  (0.83-1.07) | 0.358 | |
| Past smoker  (vs never) | 1.03  (0.99-1.06) | 0.155 | 0.97  (0.91-1.04) | 0.370 | | 0.95  (0.88-1.03) | 0.213 | 1.02  (0.87-1.19) | 0.821 | 1.05  (0.91-1.23) | 0.499 | |
| Overweight  (vs normal/under) | 0.81  (0.77-0.84) | <0.001 | 0.80  (0.75-0.87) | <0.001 | | 0.69  (0.60-0.79) | <0.001 | 0.82  (0.67-1.00) | 0.049 | 0.83  (0.67-1.03) | 0.087 | |
| Obese  (vs normal/under) | 0.63  (0.61-0.66) | <0.001 | 0.70  (0.65-0.75) | <0.001 | | 0.54  (0.47-0.61) | <0.001 | 0.66  (0.54-0.82) | 0.001 | 0.66  (0.54-0.82) | 0.001 | |
| Substance abuse | 0.84  (0.81-0.86) | <0.001 | 0.92  (0.88-0.96) | <0.001 | | 0.83  (0.77-0.89) | <0.001 | 0.90  (0.75-1.08) | 0.251 | 0.82  (0.72-0.94) | 0.003 | |
| Number of comorbidities | 1.01  (1.00-1.01) | <0.001 | 1.01  (1.00-1.01) | 0.050 | | 1.01  (1.00-1.01) | 0.773 | 1.01  (0.99-1.02) | 0.818 | 1.01  (1.00-1.02) | 0.080 | |
| PTSD | 1.05  (1.02-1.08) | <0.001 | 1.07  (1.02-1.11) | 0.002 | | 1.10  (1.03-1.18) | 0.006 | 1.05  (0.92-1.20) | 0.476 | 1.00  (0.88-1.13) | 0.950 | |
| OSA | 0.99  (0.98-1.00) | 0.010 | 0.98  (0.97-0.99) | 0.001 | | 1.00  (0.97-1.02) | 0.666 | 1.00  (0.96-1.05) | 0.985 | 0.97  (0.94-1.01) | 0.191 | |
| Diabetes | 0.94  (0.91-0.96) | 0.482 | 0.96  (0.92-1.00) | 0.384 | | 1.01  (0.95-1.08) | 0.033 | 0.99  (0.86-1.13) | 0.810 | 0.90  (0.80-1.01) | 0.927 | |
| Hyperlipidemia | 1.01  (0.98-1.05) | 0.905 | 0.98  (0.93-1.03) | 0.406 | | 0.91  (0.84-0.99) | 0.430 | 0.98  (0.93-1.16) | 0.738 | 1.01  (0.87-1.17) | 0.434 | |
| MST | 1.08  (1.01-1.15) | 0.022 | 0.95  (0.87-1.03) | 0.219 | | 1.02  (0.88-1.19) | 0.792 | 1.30  (0.90-1.87) | 0.157 | 1.08  (0.85-1.39) | 0.516 | |
| Primary care utilization | 1.00 (0.98-1.03) | <0.001 | 0.98  (0.94-1.03) | 0.012 | | 0.97  (0.91-1.04) | 0.055 | 1.03  (0.89-1.18) | 0.374 | 1.05  (0.93-1.18) | 0.268 | |
| Abbreviations: CI, confidence interval; MST, military sexual trauma; NH, non-Hispanic; OR, odds ratio; OSA, obstructive sleep apnea; PTSD, posttraumatic stress disorder; Notes: Primary care utilization was based on number of visits in the first two years of VA care.  Notes. Covariates were measured at baseline (demographics, smoking status, BMI, history of military sexual trauma) or by the first diagnosis (substance use, clinical risk factors). | | | | | | | | | | | | |

**Table S8.** Multivariable adjusted models for the effect of race and ethnicity on BP control odds at years 1, 2, and 5 – Stratified by sex

| Men | OR (95% CI) | | | | | | | | | |
| --- | --- | --- | --- | --- | --- | --- | --- | --- | --- | --- |
|  | **NH Black vs Hispanic (ref)** | **NH Black vs NH Asian** | **NH Black vs NH Other** | **NH Black vs NH White** | **Hispanic vs NH Asian** | **Hispanic vs NH Other** | **Hispanic vs NH White** | **NH Asian vs NH Other** | **NH Asian vs NH White** | **NH Other vs NH White** |
| Full model |  |  |  |  |  |  |  |  |  |  |
| Year 1 | 0.88 (0.86-0.91) | 0.85 (0.81-0.89) | 0.99 (0.95-1.04) | 0.96 (0.94-0.98) | 0.96 (0.91-1.01) | 1.12 (1.07-1.18) | 1.09 (1.06-1.11) | 1.17 (1.11-1.25) | 1.14 (1.09-1.19) | 0.97 (0.93-1.01) |
| Year 2 | 0.76 (0.74-0.79) | 0.84 (0.79-0.89) | 0.90 (0.86-0.95) | 0.86 (0.84-0.88) | 1.10 (1.04-1.17) | 1.19 (1.13-1.25) | 1.13 (1.10-1.17) | 1.08 (1.00-1.16) | 1.03 (0.97-1.09) | 0.95 (0.91-1.00) |
| Year 5 | 0.73 (0.70-0.76) | 0.74 (0.69-0.79) | 0.78 (0.74-0.83) | 0.79 (0.77-0.81) | 1.02 (0.95-1.10) | 1.08 (1.01-1.15) | 1.09 (1.05-1.13) | 1.06 (0.97-1.15) | 1.07 (1.00-1.14) | 1.01 (0.96-1.08) |
|  |  |  |  |  |  |  |  |  |  |  |
| Women | **OR (95% CI)** |  |  |  |  |  |  |  |  |  |
|  | **NH Black vs Hispanic (ref)** | **NH Black vs NH Asian** | **NH Black vs NH Other** | **NH Black vs NH White** | **Hispanic vs NH Asian** | **Hispanic vs NH Other** | **Hispanic vs NH White** | **NH Asian vs NH Other** | **NH Asian vs NH White** | **NH Other vs NH White** |
| Full model |  |  |  |  |  |  |  |  |  |  |
| Year 1 | 0.88 (0.86-0.91) | 0.85 (0.81-0.89) | 0.99 (0.95-1.04) | 0.96 (0.94-0.98) | 0.96 (0.91-1.01) | 1.12 (1.07-1.18) | 1.09 (1.06-1.11) | 1.17 (1.11-1.25) | 1.14 (1.09-1.19) | 0.97 (0.93-1.01) |
| Year 2 | 0.74 (0.68-0.80) | 1.08 (0.93-1.25) | 0.98 (0.88-1.10) | 0.84 (0.80-0.88) | 1.46 (1.25-1.72) | 1.33 (1.17-1.51) | 1.14 (1.05-1.23) | 0.91 (0.76-1.09) | 0.78 (0.67-0.90) | 0.86 (0.76-0.95) |
| Year 5 | 0.71 (0.64-0.78) | 1.01 (0.84-1.21) | 0.88 (0.77-1.01) | 0.77 (0.72-0.81) | 1.43 (1.17-1.74) | 1.24 (1.06-1.45) | 1.08 (0.98-1.19) | 0.87 (0.70-1.09) | 0.76 (0.63-0.91) | 0.87 (0.76-1.00) |

Abbreviations: CI, confidence interval; NH, non-Hispanic; OR, odds ratio;

Notes. Covariates were measured at baseline (demographics, smoking status, BMI, history of military sexual trauma) or by the first diagnosis (substance use, clinical risk factors).

**Table S9.** Multivariable models for the effects of sex and SDI on BP control odds at 1-5 years.

|  |  | | | **Year 1** | | **Year 2** | | **Year 5** | |
| --- | --- | --- | --- | --- | --- | --- | --- | --- | --- |
|  |  | | **OR (95% CI)** | | ***P*** | **OR (95% CI)** | ***P*** | **OR (95% CI)** | ***P*** |
| Women | | 1.85 (1.81-1.90) | | | <0.001 | 1.55 (1.50-1.60) | <0.001 | 1.39 (1.34-1.44) | <0.001 |
| **Demographics** | |  | | |  |  |  |  |  |
| <40 years (vs ≥40 years) | | 1.09 (1.07-1.10) | | | <0.001 | 0.98 (0.97-1.00) | 0.086 | 0.97 (0.95-0.99) | 0.003 |
| NH Black (vs NH White) | | 0.90 (0.88-0.92) | | | <0.001 | 0.83 (0.81-0.84) | <0.001 | 0.77 (0.75-0.78) | <0.001 |
| Hispanic (vs NH White) | | 1.07 (1.04-1.09) | | | <0.001 | 1.12 (1.09-1.15) | <0.001 | 1.07 (1.04-1.11) | <0.001 |
| NH Asian (vs NH White) | | 1.09 (1.04-1.14) | | | <0.001 | 1.00 (0.94-1.05) | 0.917 | 1.02 (0.95-1.09) | 0.616 |
| NH Other (vs NH White) | | 0.94 (0.90-0.98) | | | 0.002 | 0.94 (0.90-0.98) | 0.008 | 0.98 (0.93-1.03) | 0.425 |
| Not married (vs married) | | 0.89 (0.88-0.90) | | | <0.001 | 0.91 (0.90-0.93) | <0.001 | 0.89 (0.87-0.91) | <0.001 |
| **Behavioral/lifestyle factors** | |  | | |  |  |  |  |  |
| Past smoker (vs never) | | 0.88 (0.87-0.90) | | | <0.01 | 0.90 (0.88-0.91) | <0.001 | 0.91 (0.89-0.93) | <0.001 |
| Current smoker (vs never) | | 0.98 (0.96-1.00) | | | 0.029 | 0.99 (0.97-1.01) | 0.359 | 1.01 (0.98-1.04) | 0.569 |
| Overweight (vs normal/under) | | 0.78 (0.76-0.80) | | | <0.001 | 0.77 (0.75-0.79) | <0.001 | 0.80 (0.77-0.83) | <0.001 |
| Obese (vs normal/under) | | 0.63 (0.62-0.65) | | | <0.001 | 0.61 (0.59-0.63) | <0.001 | 0.64 (0.62-0.66) | <0.001 |
| Alcohol abuse or drug use (vs never) | | 0.94 (0.92-0.95) | | | <0.001 | 0.86 (0.84-0.87) | <0.001 | 0.85 (0.84-0.87) | <0.001 |
| **Clinical risk factors** | |  | | |  |  |  |  |  |
| Number of comorbidities | | 1.03 (1.03-1.04) | | | <0.001 | 0.99 (0.98-0.99) | <0.001 | 0.99 (0.98-0.99) | 0.030 |
| PTSD ever (vs never) | | 1.19 (1.18-1.21) | | | <0.001 | 1.08 (1.06-1.10) | <0.001 | 1.06 (1.04-1.08) | <0.001 |
| OSA ever (vs never) | | 1.03 (1.02-1.03) | | | <0.001 | 1.00 (1.00-1.00) | 0.163 | 1.01 (1.00-1.01) | 0.369 |
| Diabetes (vs none) | | 1.09 (1.07-1.10) | | | <0.001 | 0.99 (0.97-1.01) | 0.006 | 0.95 (0.93-0.97) | <0.001 |
| Dyslipidemia (vs none) | | 1.05 (1.03-1.08) | | | <0.001 | 0.97 (0.94-0.99) | 0.559 | 0.99 (0.96-1.02) | 0.636 |
| MST ever (vs no history) | | 1.08 (1.05-1.12) | | | <0.001 | 1.05 (1.01-1.09) | 0.013 | 1.04 (0.99-1.09) | 0.123 |
| Primary care utilization | | 1.06 (1.04-1.08) | | | <0.001 | 1.00 (0.98-1.01) | 0.0003 | 1.00 (0.98-1.02) | 0.896 |
| **Social Deprivation Index** | |  | | |  |  |  |  |  |
| Q2 vs Q1 | | 1.02 (1.00-1.04) | | | 0.084 | 1.00 (0.97-1.03) | 0.9241 | 1.03 (1.00-1.06) | 0.088 |
| Q3 vs Q1 | | 1.00 (0.98-1.03) | | | 0.932 | 1.01 (0.98-1.03) | 0.6557 | 1.03 (1.00-1.07) | 0.030 |
| Q4 vs Q1 (most vs least disadvantaged) | | 0.99 (0.97-1.01) | | | 0.392 | 0.98 (0.95-1.00) | 0.0771 | 1.02 (0.99-1.05) | 0.242 |

Abbreviations: CI, confidence interval; MST, military sexual trauma; NH, non-Hispanic; OR, odds ratio; OSA, obstructive sleep apnea; PTSD, posttraumatic stress disorder; SDI, Social Deprivation Index (Quartile 1=least disadvantaged; Quartile 4=most disadvantaged)

Notes. Primary care utilization was based on number of visits in the first two years of VA care.

**Table S10.** Patients who met criteria for hypertension with 130/80 mmHg, by sex (n=152,859).

|  | Total  (n=152,859) | Men  (n=132,293) | Women  (*n*=20,566) |
| --- | --- | --- | --- |
| BP only | 87654 (57.3) | 76936 (58.2) | 10718 (52.1) |
| Diagnosis only | 2362 (1.6) | 2164 (1.6) | 198 (1.0) |
| AHM only | 15039 (9.8) | 11181 (8.5) | 3858 (18.8) |
| Diagnosis and BP | 3812 (2.5) | 3515 (2.7) | 297 (1.4) |
| AHM and BP | 19491 (12.8) | 16386 (12.4) | 3105 (15.1) |
| AHM, diagnosis | 4959 (3.2) | 4397 (3.3) | 562 (2.7) |
| AHM, diagnosis, BP | 19542 (12.8) | 17714 (13.4) | 1828 (8.9) |

Abbreviations: AHM, antihypertensive medication; BP, blood pressure

**Table S11.** Patients who met criteria for hypertension using 130/80 mmHg, by sex, race, and ethnicity (n=152,859).

|  | Overall  (n= 152,859) | Men  (*n*=132,293) | | | | | |  | Women  (*n*=20,566) | | | | |
| --- | --- | --- | --- | --- | --- | --- | --- | --- | --- | --- | --- | --- | --- |
|  |  | **Total** | **NH White** | **NH Black** | **Hispanic** | **NH Asian** | **NH Other** | **Total** | **NH White** | **NH Black** | **Hispanic** | **NH Asian** | **NH Other** |
| BP only | 87654  (57.3) | 76936 (58.2) | 50976 (59.5) | 11388 (52.0) | 9954 (61.5) | 2128 (52.7) | 2490 (55.9) | 10718 (52.1) | 5082 (53.8) | 3445 (49.5) | 1396 (53.7) | 292 (48.8) | 503 (52.3) |
| Diagnosis only | 2362  (1.6) | 2164 (1.6) | 1431 (1.7) | 371 (1.7) | 209  (1.3) | 90 (2.2) | 63  (1.4) | 198 (1.0) | 78 (0.8) | 83  (1.2) | 21  (0.8) | 9 (1.5) | 7 (0.7) |
| Diagnosis and BP | 3812  (2.5) | 3515 (2.7) | 2155 (2.5) | 745 (3.4) | 374  (2.3) | 130 (3.2) | 111 (2.5) | 297 (1.4) | 98 (1.0) | 147 (2.1) | 33  (1.3) | 9 (1.5) | 10 (1.0) |
| AHM only | 15039  (9.8) | 11181 (8.5) | 7444 (8.7) | 1584 (7.2) | 1443 (8.9) | 344 (8.5) | 366 (8.2) | 3858 (18.8) | 1978 (20.9) | 1019 (14.6) | 568 (21.9) | 105 (17.3) | 188 (19.6) |
| AHM and BP | 19491  (12.8) | 16386 (12.4) | 10624 (12.4) | 2665 (12.2) | 1981 (12.2) | 494 (12.2) | 622 (14.0) | 3105 (15.1) | 1443 (15.3) | 1056 (15.2) | 373 (14.4) | 90 (15.0) | 143 (14.9) |
| AHM, diagnosis | 4959  (3.2) | 4397 (3.3) | 2764 (3.2) | 884 (4.0) | 461  (2.9) | 147 (3.6) | 141 (3.2) | 562 (2.7) | 217 (2.3) | 250 (3.6) | 49  (1.9) | 19 (3.2) | 27 (2.8) |
| AHM, diagnosis, and BP | 19542  (12.8) | 17714 (13.4) | 10333 (12.1) | 4259 (19.5) | 10.9 (10.9) | 702 (17.4) | 663 (14.9) | 1828 (8.9) | 549 (5.8) | 963 (13.8) | 158  (6.1) | 75 (12.5) | 83 (8.6) |

Abbreviations: AHM, antihypertensive medication; BP, blood pressure; HTN, hypertension; NH, non-Hispanic

Note: Hypertension was defined if an individual had a diagnosis code, an antihypertensive medication fill, and/or BP readings of 130/80.

**Table S12.** Demographic and clinical characteristics of the 2018-2023 cohort, by sex (n=152,859).

|  | Men  (*n*=132,293) | Women  (*n*=20,566) | *P* |
| --- | --- | --- | --- |
| Demographics |  |  |  |
| Age, y (median) | 38.9 (33.1,46.9) | 39.0 (33.5,46.2) | <0.001 |
| <40 | 72165 (54.6) | 11194 (54.4) |  |
| ≥40 | 60128 (45.5) | 9372 (45.6) |  |
| Race and ethnicity |  |  | <0.001 |
| NH White | 85727 (64.8) | 9445 (45.9) |  |
| NH Black | 21896 (16.6) | 6963 (33.9) |  |
| Hispanic | 16179 (12.2) | 2598 (12.6) |  |
| NH Asian | 4035 (3.1) | 599 (2.9) |  |
| NH Other | 4456 (3.4) | 961 (4.7) |  |
| Married | 72766 (55.8) | 8402 (41.3) | <0.001 |
| Rural | 35269 (26.7) | 4156 (20.2) | <0.001 |
| Additional health insurance | 51112 (38.7) | 7695 (37.6) | 0.001 |
| Social deprivation |  |  | <0.001 |
| Quartile 1 (least disadvantaged) | 38174 (28.9) | 5293 (25.7) |  |
| Quartile 2 | 32785 (24.8) | 5017 (24.4) |  |
| Quartile 3 | 30928 (23.4) | 5033 (24.5) |  |
| Quartile 4 (most disadvantaged) | 30406 (23.0) | 5223 (25.4) |  |
| Behavioral/lifestyle factors |  |  |  |
| Current smoker | 47307 (36.2) | 4087 (20.0) | <0.001 |
| Obese | 59291 (47.1) | 7887 (39.7) | <0.001 |
| Alcohol or drug use disorder | 25208 (19.1) | 2636 (12.8) | <0.001 |
| Clinical factors |  |  |  |
| Military sexual trauma history | 2716 (2.1) | 6875 (34.2) | <0.001 |
| PTSD | 57146 (43.2) | 9604 (46.7) | <0.001 |
| MDD | 40364 (30.5) | 9876 (48.0) | <0.001 |
| GAD | 10822 (8.2) | 3145 (15.3) | <0.001 |
| Diabetes | 8405 (6.4) | 957 (4.7) | <0.001 |
| Dyslipidemia | 42706 (32.3) | 4336 (21.1) | <0.001 |
| OSA | 35599 (26.9) | 2759 (13.4) | <0.001 |
| Number of comorbidities |  |  | <0.001 |
| 0 | 80534 (60.9) | 10574 (51.4) |  |
| 1 | 39315 (29.7) | 7430 (36.1) |  |
| 2 | 8195 (6.2) | 1825 (8.9) |  |
| ≥3 | 4249 (3.2) | 2693 (3.6) |  |
| Healthcare utilization |  |  | <0.001 |
| # primary care visits, median (IQR) | 4 (2,6) | 5 (3,8) | <0.001 |
| Follow-up period, median (IQR) | 1.8 (0.3,3.8) | 1.7 (0.2,3.8) | <0.001 |
| Baseline BP category† |  |  |  |
| >=160/100 mmHg | 4328 (3.3) | 306 (1.5) |  |
| 140-159/90-99 mmHg | 20882 (15.8) | 1534 (7.5) |  |
| 130-139/80-89 mmHg | 50524 (38.2) | 5628 (27.4) |  |
| 120-129/<80 mmHg | 23417 (17.7) | 2884 (14.0) |  |
| <120/80 mmHg | 33142 (25.1) | 10214 (49.7) | <0.001 |
| First AHM category prescribed |  |  | <0.001 |
| ACEI | 10742 (8.1) | 747 (3.6) |  |
| ARB | 4608 (3.5) | 361 (1.8) |  |
| Beta blocker | 15651 (11.8) | 4075 (19.8) |  |
| Calcium channel blocker | 7528 (5.7) | 1136 (5.5) |  |
| Diuretic | 5360 (4.1) | 2193 (10.7) |  |
| Diuretic combo with other class | 2692 (2.0) | 290 (1.4) |  |
| Other class combo (no diuretic) | 274 (0.2) | 24 (0.1) |  |
| Other medications | 2823 (2.1) | 527 (2.6) |  |

Abbreviations: ACE inhibitors, angiotensin-converting enzyme inhibitors; AHM, antihypertensive medication; ARBs, angiotensin receptor blockers; BP, blood pressure; BMI, body mass index; EHR, electronic health record; GAD, generalized anxiety disorder; MDD, major depressive disorder; MST, military sexual trauma; NH, non-Hispanic; OSA, obstructive sleep apnea; PTSD, posttraumatic stress disorder; SDI, Social Deprivation Index

Notes. Data are presented as N (%). All other data are presented as median (Q1, Q3). P-value tests differences across all sex, race, and ethnicity groups using chi-square test for categorical variables, Kruskal-Wallis test for continuous non-normally distributed variables. If an individual was in 2 distinct categories based on systolic and diastolic BP, they were placed in the higher BP category. Other medications were vasodilators, direct renin inhibitors, alpha central 2 antagonists.

**Table S13.** Multivariable models of sex and BP control at 1-5 years in the 2018-2023 cohort.

|  | |  | | **Year 1** | | | **Year 2** | | | | **Year 5** | | | |
| --- | --- | --- | --- | --- | --- | --- | --- | --- | --- | --- | --- | --- | --- | --- |
|  | |  | **OR (95% CI)** | | | ***P*** | | **OR (95% CI)** | | ***P*** | | **OR (95% CI)** | | ***P*** |
| Women | 2.42 (2.32-2.53) | | | | <0.001 | | 1.62 (1.53-1.70) | | <0.0001 | | 1.41 (1.30-1.54) | | <0.001 | |
| **Demographics** |  | | | |  | |  | |  | |  | |  | |
| <40 years (vs ≥40 years) | 0.88 (0.85-0.91) | | | | <0.001 | | 0.83 (0.80-0.87) | | <0.001 | | 0.83 (0.78-0.88) | | <0.001 | |
| NH Black (vs NH White) | 0.98 (0.94-1.02) | | | | 0.290 | | 0.92 (0.88-0.97) | | 0.001 | | 0.84 (0.78-0.90) | | <0.001 | |
| Hispanic (vs NH White) | 1.23 (1.17-1.28) | | | | <0.001 | | 1.17 (1.11-1.23) | | <0.001 | | 1.17 (1.08-1.26) | | 0.001 | |
| NH Asian (vs NH White) | 0.93 (0.85-1.02) | | | | 0.100 | | 0.97 (0.88-1.07) | | 0.554 | | 1.06 (0.91-1.23) | | 0.485 | |
| NH Other (vs NH White) | 0.95 (0.88-1.03) | | | | 0.225 | | 0.93 (0.84-1.02) | | 0.123 | | 1.02 (0.88-1.19) | | 0.760 | |
| Not married (vs married) | 0.98 (0.95-1.02) | | | | 0.307 | | 0.99 (0.96-1.03) | | 0.650 | | 0.97 (0.92-1.03) | | 0.354 | |
| **Behavioral factors** |  | | | |  | |  | |  | |  | |  | |
| Past smoker (vs never) | 0.90 (0.86-0.93) | | | | <0.001 | | 0.90 (0.86-0.94) | | <0.001 | | 0.90 (0.85-0.96) | | 0.002 | |
| Current smoker (vs never) | 0.94 (0.91-0.98) | | | | 0.005 | | 0.95 (0.91-1.00) | | 0.028 | | 0.98 (0.91-1.05) | | 0.525 | |
| Overweight (vs normal/under) | 0.65 (0.63-0.68) | | | | <0.001 | | 0.69 (0.66-0.73) | | <0.001 | | 0.70 (0.65-0.76) | | <0.001 | |
| Obese (vs normal/under) | 0.44 (0.42-0.46) | | | | <0.001 | | 0.48 (0.46-0.51) | | <0.001 | | 0.52 (0.48-0.56) | | <0.001 | |
| Alcohol abuse or drug use (vs never) | 1.13 (1.08-1.17) | | | | <0.001 | | 0.92 (0.88-0.97) | | <0.001 | | 0.99 (0.92-1.07) | | 0.841 | |
| **Clinical factors** |  | | | |  | |  | |  | |  | |  | |
| MST ever (vs no history) | 1.02 (0.97-1.08) | | | | 0.435 | | 1.00 (0.93-1.08) | | 0.939 | | 1.08 (0.97-1.22) | | 0.177 | |
| PTSD ever (vs never) | 1.44 (1.40-1.49) | | | | <0.001 | | 1.07 (1.04-1.11) | | <0.001 | | 1.10 (1.04-1.16) | | 0.001 | |
| # of comorbidities | 1.20 (1.18-1.22) | | | | <0.001 | | 1.05 (1.03-1.07) | | <0.001 | | 1.02 (0.99-1.05) | | 0.195 | |
| OSA ever (vs never) | 1.03 (1.03-1.04) | | | | <0.001 | | 1.00 (1.00-1.00) | | 0.063 | | 1.01 (1.00-1.02) | | 0.019 | |
| Diabetes (vs none) | 1.12 (1.08-1.16) | | | | 0.232 | | 0.96 (0.93-1.00) | | 0.030 | | 0.93 (0.88-0.99) | | 0.434 | |
| Dyslipidemia (vs none) | 1.04 (0.98-1.11) | | | | 0.001 | | 0.92 (0.86-0.99) | | 0.133 | | 1.04 (0.94-1.16) | | 0.765 | |
| Primary care utilization | 1.06 (1.03-1.10) | | | | <0.001 | | 0.97 (0.94-1.01) | | 0.921 | | 0.99 (0.94-1.05) | | 0.002 | |
| **Social Deprivation Index** |  | | | |  | |  | |  | |  | |  | |
| Q2 vs Q1 | 1.04 (0.99-1.09) | | | | 0.114 | | 1.03 (0.98-1.09) | | 0.187 | | 1.12 (1.04-1.21) | | 0.004 | |
| Q3 vs Q1 | 1.06 (1.02-1.11) | | | | 0.010 | | 1.02 (0.97-1.07) | | 0.469 | | 1.04 (0.96-1.12) | | 0.3401 | |
| Q4 vs Q1 | 1.17 (1.12-1.23) | | | | <0.001 | | 1.11 (1.06-1.17) | | <0.001 | | 1.17 (1.08-1.26) | | <0.001 | |

Abbreviations: CI, confidence interval; MST, military sexual trauma; NH, non-Hispanic; OR, odds ratio; OSA, obstructive sleep apnea; PTSD, posttraumatic stress disorder; SDI (Quartile 1=least disadvantaged; Quartile 4=most disadvantaged)

Notes. Primary care utilization was based on number of visits in the first two years of VA care.

| **Table S14.** BP control at 1-5 years in the 2018-2023 cohort, by sex, race, and ethnicity. | | | | | | | |  |  |
| --- | --- | --- | --- | --- | --- | --- | --- | --- | --- |
| *Within Sex by Race and Ethnicity* | | **OR (95% CI)** | | | | ***P*** | | |  |
| **Year 1** | |  | | | |  | | |  |
| NH White (Women vs. Men) | | 2.56 (2.40-2.72) | | | | <0.001 | | |  |
| NH Black | | 2.20 (2.03-2.38) | | | | <0.001 | | |  |
| Hispanic | | 2.40 (2.14-2.69) | | | | <0.001 | | |  |
| NH Asian | | 2.01 (1.57-2.59) | | | | <0.001 | | |  |
| NH Other | | 2.99 (2.41-3.69) | | | | <0.001 | | |  |
| **Year 2** | |  | | | |  | | |  |
| NH White (Women vs. Men) | | 1.63 (1.56-1.70) | | | | <0.001 | | |  |
| NH Black | | 1.48 (1.41-1.56) | | | | <0.001 | | |  |
| Hispanic | | 1.61 (1.45-1.78) | | | | <0.001 | | |  |
| NH Asian | | 1.22 (0.99-1.50) | | | | 0.067 | | |  |
| NH Other | | 1.58 (1.35-1.86) | | | | <0.001 | | |  |
| **Year 5** | |  | | | |  | | |  |
| NH White (Women vs. Men) | | 1.46 (1.29-1.66) | | | | <0.001 | | |  |
| NH Black | | 1.40 (1.20-1.63) | | | | <0.001 | | |  |
| Hispanic | | 1.34 (1.05-1.70) | | | | <0.001 | | |  |
| NH Asian | | 1.12 (0.66-1.87) | | | | 0.740 | | |  |
| NH Other | | 2.11 (1.35-3.29) | | | | 0.002 | | |  |
|  | | | |  | |  | | |  |
| *Within Race and Ethnicity by Sex* | | | **Men** | | | | **Women** | | |
|  |  | | **OR (95% CI)** | | **OR (95% CI)** | | | |  |
| **Year 1** | |  | | |  | | | |  |
| NH Black (vs. NH White) | | 1.03 (0.98-1.08) | | | 0.86 (0.80-0.92) | | | |  |
| Hispanic (vs. NH White) | | 1.22 (1.16-1.28) | | | 1.16 (1.05-1.28) | | | |  |
| NH Asian (vs. NH White) | | 0.99 (0.90-1.10) | | | 0.72 (0.59-0.88) | | | |  |
| NH Other (vs. NH White) | | 0.92 (0.83-1.01) | | | 0.98 (0.84-1.15) | | | |  |
| **Year 2** | |  | | |  | | | |  |
| NH Black (vs. NH White) | | 0.96 (0.91-1.01) | | | 0.84 (0.77-0.93) | | | |  |
| Hispanic (vs. NH White) | | 1.18 (1.11-1.25) | | | 1.14 (1.00-1.30) | | | |  |
| NH Asian (vs. NH White) | | 0.98 (0.88-1.09) | | | 0.93 (0.72-1.20) | | | |  |
| NH Other (vs. NH White) | | 0.93 (0.83-1.03) | | | 0.93 (0.76-1.14) | | | |  |
| **Year 3** | |  | | |  | | | |  |
| NH Black (vs. NH White) | | 0.85 (0.79-0.92) | | | 0.76 (0.66-0.89) | | | |  |
| Hispanic (vs. NH White) | | 1.15 (1.06-1.26) | | | 1.14 (0.92-1.40) | | | |  |
| NH Asian (vs. NH White) | | 1.09 (0.93-1.28) | | | 0.82 (0.54-1.24) | | | |  |
| NH Other (vs. NH White) | | 0.99 (0.84-1.17) | | | 1.14 (0.82-1.59) | | | |  |
| Abbreviations: BP, blood pressure; CI, confidence interval; NH, non-Hispanic; OR, odds ratio;  Notes. Covariates were measured at baseline (demographics, smoking status, BMI, history of military sexual trauma) or by the first diagnosis (substance use, clinical risk factors). | | | | | | | | |  |

| **Table S15.** BP control at 1-5 years in the 2018-2023 cohort, by sex, race, and ethnicity. | | | | | | | |  |  |
| --- | --- | --- | --- | --- | --- | --- | --- | --- | --- |
| *Within Sex by Race and Ethnicity* | | **OR (95% CI)** | | | | ***P*** | | |  |
| **Year 1** | |  | | | |  | | |  |
| NH White (Women vs. Men) | | 1.96 (1.89-2.04) | | | | <0.001 | | |  |
| NH Black | | 1.74 (1.67-1.81) | | | | <0.001 | | |  |
| Hispanic | | 2.07 (1.90-2.26) | | | | <0.001 | | |  |
| NH Asian | | 1.26 (1.07-1.49) | | | | <0.001 | | |  |
| NH Other | | 1.78 (1.56-2.04) | | | | <0.001 | | |  |
| **Year 2** | |  | | | |  | | |  |
| NH White (Women vs. Men) | | 1.63 (1.56-1.70) | | | | <0.001 | | |  |
| NH Black | | 1.48 (1.41-1.56) | | | | <0.001 | | |  |
| Hispanic | | 1.61 (1.45-1.78) | | | | <0.001 | | |  |
| NH Asian | | 1.22 (0.99-1.50) | | | | <0.001 | | |  |
| NH Other | | 1.58 (1.35-1.86) | | | | <0.001 | | |  |
| **Year 5** | |  | | | |  | | |  |
| NH White (Women vs. Men) | | 1.47 (1.40-1.56) | | | | <0.001 | | |  |
| NH Black | | 1.38 (1.30-1.46) | | | | <0.001 | | |  |
| Hispanic | | 1.43 (1.26-1.62) | | | | <0.001 | | |  |
| NH Asian | | 0.88 (0.68-1.14) | | | | 0.340 | | |  |
| NH Other | | 1.13 (0.93-1.39) | | | | 0.215 | | |  |
|  | | | |  | |  | | |  |
| *Within Race and Ethnicity by Sex* | | | **Men** | | | | **Women** | | |
|  |  | | **OR (95% CI)** | | **OR (95% CI)** | | | |  |
| **Year 1** | |  | | |  | | | |  |
| NH Black (vs. NH White) | | 0.96 (0.94-0.98) | | | 0.84 (0.80-0.88) | | | |  |
| Hispanic (vs. NH White) | | 1.09 (1.06-1.11) | | | 1.14 (1.05-1.23) | | | |  |
| NH Asian (vs. NH White) | | 1.14 (1.09-1.19) | | | 0.78 (0.67-0.90) | | | |  |
| NH Other (vs. NH White) | | 0.97 (0.93-1.01) | | | 0.86 (0.76-0.95) | | | |  |
| **Year 2** | |  | | |  | | | |  |
| NH Black (vs. NH White) | | 0.86 (0.84-0.88) | | | 0.77 (0.72-0.81) | | | |  |
| Hispanic (vs. NH White) | | 1.13 (1.10-1.17) | | | 1.08 (0.98-1.19) | | | |  |
| NH Asian (vs. NH White) | | 1.03 (0.97-1.09) | | | 0.76 (0.63-0.91) | | | |  |
| NH Other (vs. NH White) | | 0.95 (0.91-1.00) | | | | | 0.87 (0.76-1.00) | |  |
| **Year 5** | |  | | | | |  | |  |
| NH Black (vs. NH White) | | 0.79 (0.77-0.81) | | | 0.70 (0.66-0.75) | | | |  |
| Hispanic (vs. NH White) | | 1.09 (1.05-1.13) | | | 1.04 (0.93-1.17) | | | |  |
| NH Asian (vs. NH White) | | 1.07 (1.00-1.14) | | | 0.67 (0.54-0.84) | | | |  |
| NH Other (vs. NH White) | | 1.01 (0.96-1.08) | | | 0.79 (0.67-0.94) | | | |  |
| Abbreviations: BP, blood pressure; CI, confidence interval; NH, non-Hispanic; OR, odds ratio;  Notes. Covariates were measured at baseline (demographics, smoking status, BMI, history of military sexual trauma) or by the first diagnosis (substance use, clinical risk factors). | | | | | | | | |  |
